# Thermo-economic and environmental optimization using PSO of solar organic Rankine cycle with flat plate solar collector

**DOI:** 10.1016/j.heliyon.2023.e13697

**Published:** 2023-02-21

**Authors:** Guillermo Valencia Ochoa, Eunice Villicaña Ortiz, Jorge Duarte Forero

**Affiliations:** a Department of Mechanical Engineering, Universidad del Atlántico, Barranquilla, 081007, Colombia; b Energy Engineering, Universidad de Ingeniería y Tecnología (UTEC), Lima, 15063, Peru; c Engineering Faculty, Mechancial Engineering Department, Universidad del Atlántico, Carrera 30, Número 8–49, Puerto Colombia, Barranquilla, 080007, Colombia

**Keywords:** Thermo-economic, Solar organic Rankine cycle, Flat-plate collector, PSO optimization, Environmental impact, Hourly simulations

## Abstract

The use of solar energy is considered a potential strategy for the production of electrical energy through thermal heat sources. This article portrays a study framed to be energetic, economic, and environmental fields. This study was carried out in two thermal configurations: the Regenerative Rankine Cycle (RORC) and the Simple Organic Rankine Cycle (SORC), which use solar energy to supply electrical power to a building. The thermodynamic and economic models were proposed for each subsystem of the thermal process, allowing hourly simulations to know the economic indicators such as the payback period (PBP), the levelized cost of energy (LCOE), the specific investment cost (SIC), and the initial investment cost ($C_{Inv}$). The effect of operational variables such as the pressure ratio (rp), the evaporator pinch point temperature (Ap), the condensation pinch point temperature (Tcond), and the solar collector area (Ac) on the Relative Annual Benefit (RAB) were studied. Finally, the Particle Swarm Optimization (PSO) algorithm was implemented to optimize the economic indicators and the environmental impact of the thermal configurations. Results showed that the RORC configuration presented a better performance in terms of generation, purchase, and hourly sale of energy. However, in terms of RAB, the SORC (39,833 USD/year) showed better results in contrast to the RORC (39,604 USD/year) for an evaporator pinch point temperature of 35 °C. Finally, the application of the PSO optimization algorithm allowed the reduction of the LCOE (11.64%), SIC (11.67%), and PBP (11.81%) thermo-economic indicators from the base condition for the SORC, and the reductions obtained in the RORC were LCOE (18.11%), SIC (10.67%), and PBP (11.11%). However, the decrease in environmental Impact for both systems was less than 1% as a consequence of the high contribution of thermal oil in the construction phase of the system.

## Highlights

• Thermo-economic and environmental assessments of two solar ORC configurations are evaluated in the most solar potentials in Colombia.
• The evaluation of potential environmental impact improvement is conducted for two ORC configurations with flat-plate collector solar.
• An hourly parametric study is presented considering the energy, thermo-economic, and environmental parameters.
• Thermo-economic and environmental performance parameters were minimized using the PSO method for the two solar ORC configurations.

## 1 Introduction

In recent years, aspects related to the use of renewable and clean energy sources have taken great importance due to the depletion of energy resources, pollution issues, and climate change [1,2]. Renewable energy sources are considered sustainable and environmentally friendly because their ecological footprint is lower than conventional energy systems [3]. The energy obtained from the sun calculated through different models is a limitless source that is available to be exploited through solar collectors [4], which allow the transformation of solar radiation into thermal energy for energy generation systems [5]. Among the different energy generation systems, there are the organic Rankine cycles (ORC), which are an attractive option to be coupled to solar collectors and to operate efficiently with energy and economic benefits, reducing greenhouse gas emissions due to the high primary energy consumption [6].

Studied from different approaches, the organic Rankine cycles, energetically fed by solar collectors have been extensively researched. The first scenario includes studies oriented toward energy and exergy analysis, such as the one carried out by Wang et al. [7], who analyzed the behavior of a solar plant using a parabolic collector (PC) connected uniquely to a simple organic Ranking Cycle (SORC), under one year of transient operating conditions for three different regions. Baccioli et al. [8] studied the energetic and exergetic of a small scale SORC, considering three types of unconcentrated collectors: a compound parabolic concentrator (CPC), an evacuated tube collector, and a flat-plate collector. Ashouri et al. [9] performed the recuperative organic Rankine cycle (RORC) energy analysis using pentane, R245fa, toluene, and R134a as working fluids. Arteconi et al. [10] investigated the electrical and thermal performance changes of an ORC with a thermal storage tank.

In a second scenario, there are studies related to the organic working fluids selection, which is a decisive step in the performance of the ORC. In this context, research made by Ustaoglu et al. [11] studied the performance of wet, dry, and isentropic fluids from the energy and exergy points of view. Moreover, Ashouri et al. [12] investigated the thermodynamic performance of a RORC connected to a parabolic trough solar collector (PTCs). Tiwari et al. [13] developed energy, exergy analysis and a parametric optimization of a solar ORC, considering mixtures of isohexane/pentane, hexane/pentane, and butane/pentane. The results depicted the use of a butane/pentane mixture in the ORC, generating an energy efficiency of 9.92% with 0.56/0.44 and an exergetic efficiency of 46.98% (0.556/0.444). As for the multi-objective optimization, the exergy efficiency improved by 19.90% for the butane/pentane mixture, the latter being the best-working fluid among those considered in the study. However, the use of these azeotropic mixtures implies a low heat transfer coefficient, which results in a larger heat exchange area, leading to higher purchase equipment costs. Therefore, evaluating the economic impact of the use of this type of mixture is an aspect to be considered.

Garg & Orosz [14] performed the optimization of an ORC using pure fluids and azeotropic mixtures for solar and waste heat applications, all using Particle Swarm Optimization (PSO). In this paper, the authors proposed a new function defined as the ratio between the specific investment cost and the effective heat transfer. The results depicted that the R134a and the R152a were the fluids with the highest enthalpy recovery from the thermal source at the lowest investment cost. In addition, the authors concluded that the choice of working fluids in the SORC is less relevant compared to the waste heat recovery systems (WHR). However, the authors only considered a single objective function and did not assess the recovery period of the system. Desai and Bandyopadhyay [15] proposed a methodology based on thermo-economic parameters (LCE) for the selection of working fluids through a new graphic representation called a working fluid selection diagram. The proposal also included the selection between parabolic trough and Fresnel collector solar. The authors manifested that the selection between the steam Rankine cycle (SRC) and the ORC is influenced by the type of solar collector, and the system investment cost obtained with a parabolic trough collector was 0.344 $/kWh and 0.364 $/kWh for the R113 and isohexane, respectively. However, the economic inidicator using a linear Fresnel was SRC (0.422 $/kWh), which was higher than the parabolic trough collector solar (0.353 $/kWh).

The analysis of the ORC thermo-economic performance is a fundamental step that allows evaluating of the viability of implementing this type of technology. In this context, the study made by Spayde et al. [16] presented an analysis to determine the economic, energy, and environmental benefits of implementing a solar ORC with power storage (EES) using R-236a as a working fluid to supply electricity to different types of commercial buildings. The results revealed that the proposed system ORC-EES was able to satisfy 42% of the total building's energy demand. Besides, in environmental terms, the authors found a reduction in carbon dioxide emissions (CDE) of 283,219 kg/year. Garcia-Saez et al. [17] evaluated, technically and economically, the integration of a solar system and ORC to supply a significant amount of energy requirements in residential areas. In this research, the system was the result of combining a SORC, heat, and power (CHP), and a reversible solar HP system. The results found out that the reversible solar HP system was more economically profitable than the solar system ORC-CHP with a recovery period (PBP) of 3.3 years. Also, this last system achieved an 80 °C energy production. In this study, the authors only considered the PBP in economic terms, without considering other significant parameters.

Cioccolanti et al. [18] evaluated the life cycle assessment of a small-scale ORC trigeneration system. The system was composed of 50 m^2^ compound parabolic collector (CPC) solar field, a 3 m^3^ thermal storage tank with a generation of 3.5 kW ORC, and 17 kW for the absorption chiller. The results revealed that the R245fa allowed environmental improvements compared to R245ca. Researchers also added that the increment of the solar field from 50 m^2^ to 100 m^2^ brought a reduction of the environmental Impact. Ramos et al. [19] considered two flat-plate solar collector systems (FPC-ORC) and an evacuated-tube collector-ORC (ETC-ORC). In this study, the researchers developed an optimization model able to maximize the system's electrical power, taking into account the operational limits of the solar collector. The results depicted that with an area of 60 m^2,^ the FPC-ORC system had a production of 460 W, using R245fa as the working fluid; while the configuration ETC-ORC was more powerful than 1700 using R1233zd as the working fluid.

Bellos & Tzivanidis [20] optimized a solar-trigeneration system in energy and exergy terms. Parabolic trough collector solar (PTCs) was used to supply the input of heat demand with an area of 1000 m^2^. The system consisted of a thermal storage tank, an ORC, and a heat absorption pump with LiBr–H2O, considering six work fluids. According to the results, toluene was the one with the highest output efficiency in the base condition (27.97%). Petrollese & Cocco [21] minimized the LCOE of a solar-based ORC power plant. The authors first studied the influence of the heat transfer fluid mass flow, its inlet temperature, and the ambient air temperature separately. After, they evaluated the influence of these parameters simultaneously, considering three working fluids. The results revealed that considering multiple scenarios leads to an ORC lower performance but less sensitive to the variations in the input parameters system.

The fluctuating characteristics of solar radiation restrict energy conversion efficiency and thermal SORC performance [22,23]. In this context, the research done by Li and Li [24] carried out a dynamic optimization of a small-scale solar organic Rankine cycle (s-ORC) from a thermo-economic point of view using typical one-year radiation data (TSRY) for different zones. In this study, the researchers implemented a multi-objective genetic algorithm (GA), considering the evaporation temperature and the thermal storage capacity as decisive variables. The authors concluded that a minimum scale of the solar-ORC is profitable for a given location. Also, the rate of growth in profitability increases as the system scale increases. Similar studies were presented by Hajabdollahi et al. [25], which led to an optimization of a solar regenerative organic Rankine cycle. In this study, the authors defined the relative annual benefit (RAB) as an objective function to be maximized by the Real Parameters Genetic Algorithm (RPGA) using three working fluids (R123, R245fa, and isobutane). The results of the optimization revealed that the isobutane had the best thermo-economic performance (258810 $/year), followed by the R245fa (68173 $/year).

Therefore, the main contribution of this article is to present a thermo-economic and environmental optimization of two organic cycle systems using a flat-plate solar collector. The study was done considering the solar radiation data from a specific area of Colombia due to its high solar potential. An hourly parametric study is presented considering the key energy, thermo-economic, and environmental parameters such as the Payback Period (PBP), the Specific Investment Cost (SIC), the environmental impact of both systems, and the Levelized Cost of Energy (LCOE).

## 2 Methodology

### 2.1 System description

**Fig. 1:**
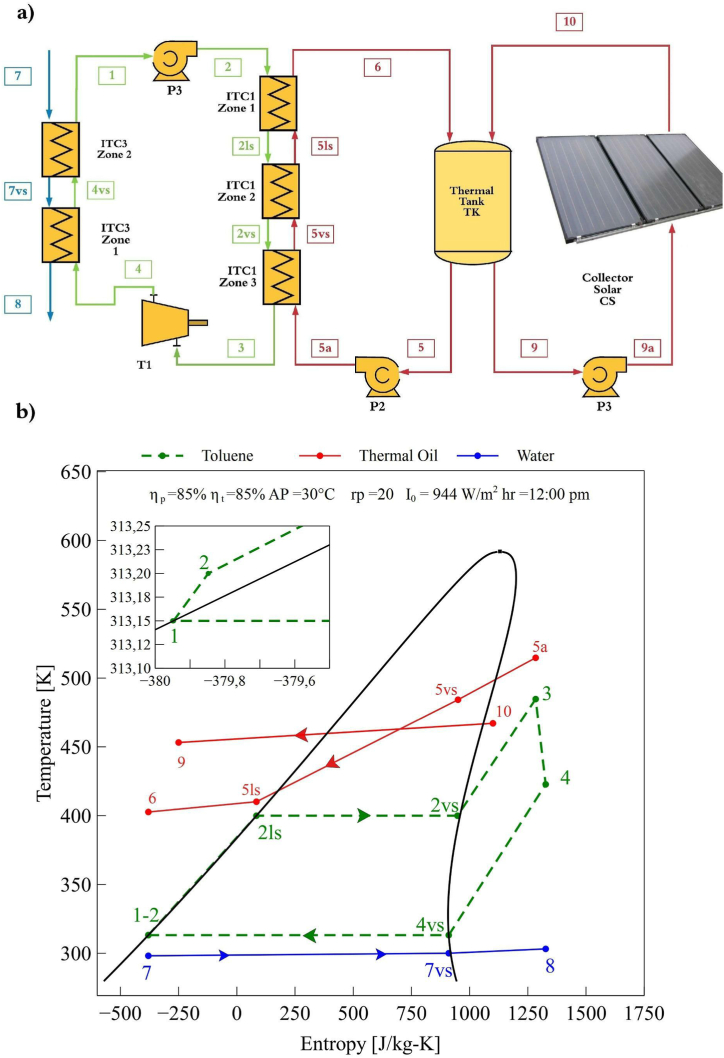
Solar -SORC configuration, a) physical structure, and b) T-S diagram.

Fig. 1 shows the physical structure of the solar SORC and the T-s diagram. In this thermal process, the organic working fluid used was toluene due to its good thermo-economic performance, according to the studies shown by Wang et al. [26]. Initially, the solar radiation affects the solar surface causing the temperature increase of the input flow (Point 9a) to the output flow (Point 10), as shown in Fig. 1a. The pump (P1) drives the thermal oil to help its ascent to the solar collector, as these are normally inclined about 30–50°. This process is carried out continuously during the 24-h of a day, which is the time considered. On the other hand, the output flow from the tank (point 5) is driven by pump 2 (P2) and then fed into the evaporator (ITC1). The evaporator is divided into three zones, including the heating zone (Zone 1), the evaporation area (Zone 2), and the overheating zone (Zone 3). In the first stage, the fluid starts from a saturated liquid at a temperature of 40 °C and a pressure of 157.85 kPa (Point 2), as shown in Fig. 1b. Then in Zone 1, the fluid is preheated to its critical temperature in the process 2-2ls.

Subsequently, in the process 2ls-2vs (Zone 2), the fluid absorbs the heat from the thermal fluid, causing a phase change at a constant temperature to saturated steam, as shown in Fig. 1b. Finally, in zone three (Zone 3), the fluid passes from saturated steam to superheated steam in process 2vs-3. Throughout this process, the thermal fluid is kept in the liquid phase due to its high thermal stability. The evaporator output stream (point 3) feeds the turbine, where it expands to a low-pressure thermodynamic state (point 4), generating output energy coupled with the generator. Then, to close the thermal cycle, the output steam from the turbine is supplied to the condenser heat exchanger (ITC3). In the first zone (Zone 1), the steam passes from superheated to saturated steam (process 4-4vs), using the cooling water as a heat sink. Successively, the steam condenses at a constant temperature in process 4vs-1 (Zone 2). Finally, the fluid exists as a saturated liquid at low-pressure, and then the cycle is performed again.

**Fig. 2:**
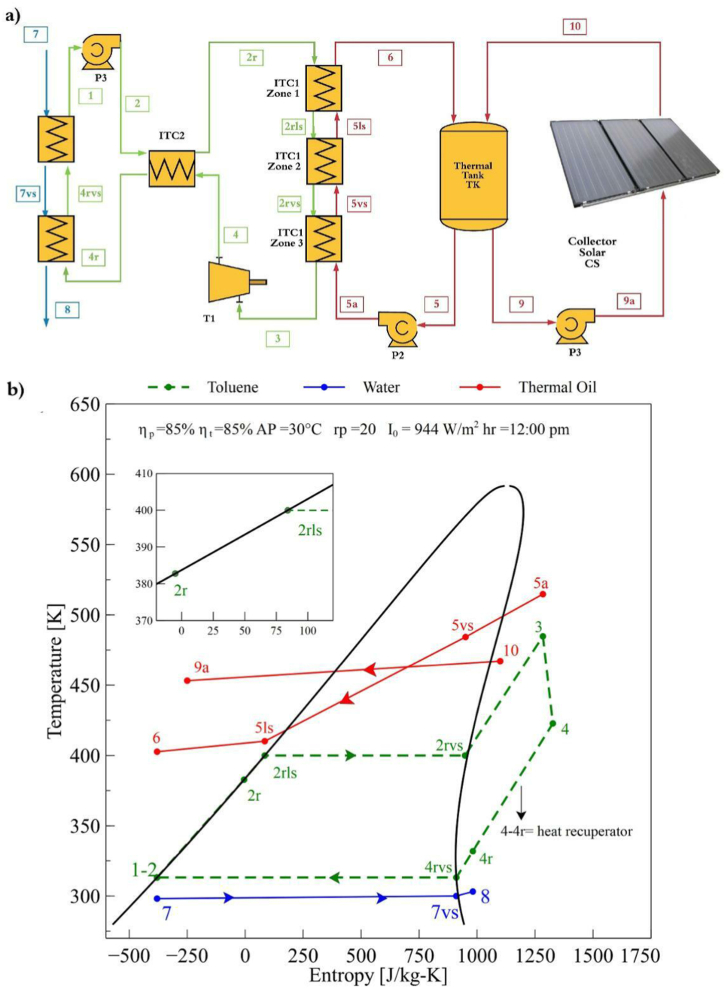
Solar -RORC configuration, a) physical structure, and b) T-S diagram.

Fig. 2a and b shows the physical structure of the Solar-RORC, which is similar to the SORC configuration described. However, the Solar-RORC configuration has a heat recovery system (ITC2), which is a plate heat exchanger. According to Fig. 2b, the internal heat of the cycle contained in the turbine exhaust steam is used to increase the temperature in the fluid preheating zone (process 2-2r). In this sense, it is possible to reduce the heat from the thermal source and increase the system's thermal efficiency.

### 2.2 Description of the place

**Fig. 3:**
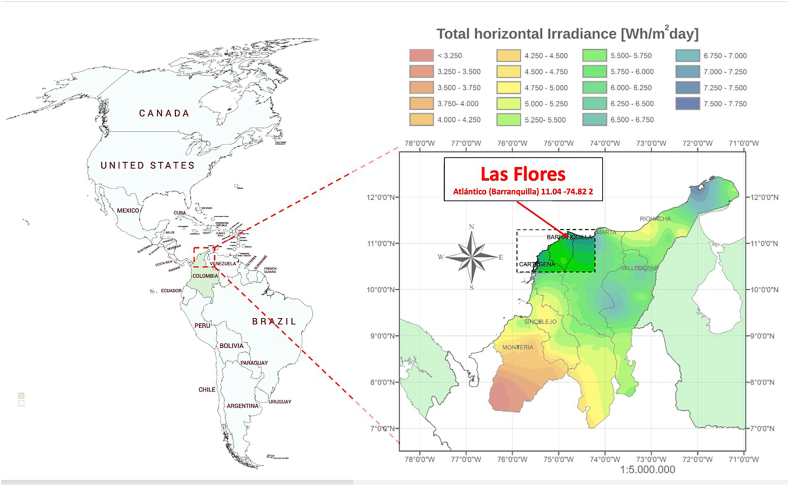
Location of the place considered in the study.

Fig. 3 shows the place under study called las Flores located in Barranquilla city in the department of Atlántico, where the average annual irradiance value is 6500 Wh/m^2^ day.

### 2.3 ORC modeling

The exergy and energy analyses were carried out based on the first and second thermodynamic balance applied to each component of the thermal process, which are considered as a control volume system. The system is an open system with isolated components; also, the changes in power and kinetic energy were neglected [27,28]. The mass balance is expressed according to equation (1).

(1) $$\sum {\dot{m}}_{in}-\sum {\dot{m}}_{out}=0$$

where ${\dot{m}}_{in}$ is the input mass flow and ${\dot{m}}_{out}$ is the output mass flow in kg/s. Similarly, the energy balance is governed by equation (2).

(2) $${\dot{Q}}_{cv}-{\dot{W}}_{cv}+\sum {\dot{m}}_{in}∙h_{in}-\sum {\dot{m}}_{out}∙h_{out}=0$$

where ${\dot{Q}}_{cv}$ is the heat transfer power in kW, ${\dot{W}}_{cv}$ is the work done on the open system, $\dot{m}$ and h represents the mass flow in kg/s and the enthalpy in kJ/kg-K, respectively. Table 1 shows the equations obtained from the energy balance by component of the SORC and RORC configuration.

**Table 1:** First law of thermodynamic applied to each component of the process.

System	Component	Equation
SORC/RORC	ITC1	${\dot{Q}}_{ITC1}={\dot{m}}_{2r}∙\left(h_{3}-h_{2r}\right)$
RORC	ITC2	${\dot{Q}}_{ITC2}={\dot{m}}_{2r}∙\left(h_{2r}-h_{2}\right)$
SORC/RORC	ITC3	${\dot{Q}}_{ITC3}={\dot{m}}_{4r}∙\left(h_{4r}-h_{1}\right)$
SORC/RORC	T1	${\dot{W}}_{T1}={\dot{m}}_{3}∙\left(h_{3}-h_{4}\right)$
SORC/RORC	P1	${\dot{W}}_{P1}={\dot{m}}_{9}∙\left(h_{9a}-h_{9}\right)$
SORC/RORC	P2	${\dot{W}}_{P2}={\dot{m}}_{5}∙\left(h_{5a}-h_{5}\right)$
SORC/RORC	P3	${\dot{W}}_{P3}={\dot{m}}_{2}∙\left(h_{2}-h_{1}\right)$
SORC/RORC	Collector Solar (CS)	${\dot{Q}}_{CS}=I_{0}\cdot \eta_{c}{\cdot A}_{c}$
SORC/RORC	Thermal storage (TK)	${\dot{Q}}_{TK}=m_{at}\cdot {Cp}_{at}\left(T_{6}-T_{5}\right)$

The specific physical exergy for the fluid flow is calculated from equation (3) [29].

(3) $$e=\left(h-h_{0}\right)-T_{0}∙\left(s-s_{0}\right)$$

where s and h are the specific entropy and enthalpy, respectively. The subscript indicates the reference conditions (25 °C and 101.23 kPa).

Equation (4) was used to calculate the exergy destroyed by each component.

(4) $${\dot{E}}_{D}=\sum_{in}\dot{m}∙e-\sum_{out}\dot{m}∙e+\sum_{in}{\dot{E}}_{Q}-\dot{W}$$

where $\dot{W}$ is the exergy by mechanical work on the device and ${\dot{E}}_{Q}$ is the exergy by heat transfer.

The total power generated by the system is expressed according to equation (5)

(5) $${\dot{W}}_{net}={\dot{W}}_{T}-\sum {\dot{W}}_{P}$$

where ${\dot{W}}_{t}$ and ${\dot{W}}_{p}$ are the power in the turbine and pumps, respectively. For the modeling of this work, an ideal energy transformation process was considered from the mechanical to the electrical convertion. That is, the conversion efficiency was 100%

#### 2.3.1 Heat exchanger modeling: evaporator, condenser, and regenerator

For the evaporator heat exchanger analysis, the equipment was divided into three zones according to the process that takes place in the organic working fluid along with the heat exchanger: the preheating, the evaporation, and the superheating. The associated heat rate in the three zones is calculated using equations (6), (7), (8).

(6) $${\dot{Q}}_{Pre}={\dot{m}}_{wf}∙\left(h_{2rls}-h_{2rvs}\right)$$

(7) $${\dot{Q}}_{Evap}={\dot{m}}_{wf}∙\left(h_{2r}-h_{2rvs}\right)$$

(8) $${\dot{Q}}_{Sob}={\dot{m}}_{wf}∙\left(h_{2rvs}-h_{3}\right)\text{,}$$

where ${\dot{m}}_{wf}$ is the mass flow rate of the working fluid. The heat transfer area is given by equation (9).

(9) $$A=\dot{Q}/\;U{∙\Delta T}_{ml}$$

where U is the overall heat transfer coefficient calculated considering a thermal resistance circuit in series from the hot fluid to the cold fluid, given by equation (10).

(10) $$\frac{1}{U}=\frac{1}{h_{to}}+R_{w}+\frac{1}{h_{wf}}$$

where $h_{to}$ is the convective heat transfer coefficient for the thermal oil side, $R_{w}$ is the wall resistance, $h_{wf}$ is the heat transfer coefficient for the working fluid. The heat transfer area is calculated as the sum of the areas required for each phase, according to equation (11).

(11) $$A=A_{Pre}+A_{Evap}+A_{Sob}$$

The evaporator plates number is calculated as follows, equation (12).

(12) $$N_{p}=A_{Pre}/\left(L∙W\right)+A_{Evap}/\left(L∙W\right)+A_{Sob}/\left(L∙W\right)$$

where L and W are the height and width of the plates, respectively. The heat transfer coefficient of the working fluid and thermal oil for the single-phase are shown in equation (13) [30].

(13) $$Nu=\frac{h∙D_{h}}{k}=0.78{∙R}_{e}^{0.5}{∙P}_{r}^{1/3}$$

where h is the heat transfer coefficient, $D_{h}$ is the hydraulic diameter, k is the thermal conductivity, $R_{e}$ is the Reynolds number, and $P_{r}$ is the Prandtl number.

For the two-phase region, the working fluid's heat transfer coefficient was modeled according to equation (14) [31].

(14) $$Nu=\frac{h∙D_{h}}{k}=0.00187∙{\left(\frac{q∙d_{0}}{k_{f}}\right)}^{0.56}∙{\left(\frac{d_{0}∙h_{fg}}{\alpha_{i}^{2}}\right)}^{0.31}∙P_{r}^{0.35}$$

where q is the heat flux ($W\;m^{-1}$), $d_{0}$ in the bubble departure diameter (m), $k_{f}$ is the liquid-phase thermal conductivity ($Wm^{-1}\;K^{-1}$), $h_{fg}$ is the latent heat of evaporation (${J\;kg}^{-1}$), and $\alpha_{i}$ is the termal diffusivity ($m^{2}\;S^{-1}$).

Besides, the condenser heat exchanger was divided into two zones. The cooling zone and the condensing zone, and the heat in each of the zones are given by equations (15), (16).

(15) $${\dot{Q}}_{Cool}={\dot{m}}_{wf}∙\left(h_{4r}-h_{4rvs}\right)$$

(16) $${\dot{Q}}_{Cond}={\dot{m}}_{wf}∙\left(h_{4rvs}-h_{1}\right)$$

The overall heat transfer coefficient is given by equation (17).

(17) $$\frac{1}{U}=\frac{1}{h_{rf}}+R_{w}+\frac{1}{h_{wf}}$$

where $h_{rf}$ is the heat transfer coefficient on the cold side, $R_{w}$ is the wall resistance, and $h_{wf}$ is the convective heat transfer coefficient on the working fluid side. The condenser heat transfer area is calculated as the sum of its areas, according to equation (18).

(18) $$A=A_{Cool}+A_{Cond}$$

The plates number in the condenser heat exchanger is calculated as follows, equation (19).

(19) $$N_{p}=A_{Cool}/\left(L∙W\right)+A_{Cond}/\left(L∙W\right)$$

where L and W are the height and width of the plate, respectively. Nusselt number correlation used for the condensing and cooling phases are shown in equations (20), (21) [32,33].

(20) $${Nu}_{Cond}=4.18{∙R}_{eq}^{0.4}{∙P}_{r}^{0.3}$$

(21) $${Nu}_{Cool}=0.78{∙R}_{e}^{0.5}{∙P}_{r}^{1/3}$$

where $R_{eq}$ is the Reynolds number for the equivalent mass expenditure, and $P_{r}$ is the Prandtl number.

Finally, for the area calculation of the heat recovery area (ITC2), the energy transfer of sensible heat is taken into account, the LMTD method was used, and the U was done using equations (17), (18), considering only the cooling stage.

#### 2.3.2 Solar collector modeling

This study considered flat-plate collector solar (evacuated, selective surface type A) as a heat source coupled to the proposed configurations. The modeling of this system was done according to the approach presented by Kerme et al. [29], in which the useful heat of collector gain can be calculated by equation (22).

(22) $${\dot{Q}}_{col}=I_{0}{\cdot A}_{c}\cdot \;\left[F_{R}\cdot \alpha \cdot \tau -F_{R}\cdot U_{L}\cdot \frac{\left(T_{oil,in}-T_{a}\right)}{I_{0}}\right]$$

where $I_{0}$ is the total solar irradiance incident on the collector surface in $W/m^{2}$, $A_{c}$ is the collector area in $m^{2}$ which was set at 200 m^2^, (τ) is the transmittance, and (α) is the absorbance (α=0.70) [34]. Similarly, the term $\left(F_{R}\cdot U_{L}\right)$ is the heat removal factor multiplied by the overall heat transfer coefficient, whose value was 3.3 W/m^2^ °C [34], $T_{a}$ is the reference temperature, $T_{oil,in}$ is the thermal oil inlet temperature to the solar collector (180 °C), and $T_{s,i}$ is the initial temperature of the thermal storage tank, which was considered at 250 °C, with an assumed thermal storage tank volume was 50 m^3^ [28].

On the other hand, the term ($I_{0}$) in equation (22) is the hourly radiation incident on the collector surface according to equation (23) [35].

(23) $$I_{o}=I_{b}{∙\chi }_{b}+I_{d}∙\chi_{d}+\left(I_{b}+I_{d}\right){∙\chi }_{r}$$

where $I_{b}$, and $I_{d}$ are the hourly beam and diffuse radiations on the collector surface, $\chi_{d}$ is the tilt factor calculated according to equation (24) [35].

(24) $$\chi_{b}=\frac{\sin\;\delta ∙\sin\left(\varphi -\beta \right)+\sin\;\delta ∙\cos\;w∙\cos\left(\varphi -\beta \right)}{\sin\;\varphi ∙\sin\;\delta +\cos\;\varphi ∙\cos\;\delta ∙cosw}$$

where φ is the latitude angle, δ is the solar declination angle, β is the tilt angle of the collector, and w is the solar hour. The solar declination angle (δ), is calculated using equation (25).

(25) $$\delta =23.45∙\sin\left(360∙\frac{284+n}{365}\right)$$

where n is the julian day. Finally, the tilt factor of diffuse radiation ($\chi_{d}$) and reflected ($\chi_{r}$) radiation [36] are given by equations (26), (27).

(26) $$\chi_{d}=\frac{1+\cos\;\beta }{2}$$

(27) $$\chi_{r}=0.2∙\left(\frac{1-\cos\;\beta }{2}\right)$$

#### 2.3.3 Thermal storage system

The thermal system modeled in this work contains a thermal storage tank to guarantee the operability of the system in hours in the absence of solar radiation. The tank was modeled in a stratified way, assuming uniform mixing, and the variation of the tank outlet temperature over time is obtained according to equation (28).

(28) $${\left({\dot{m}}_{to}{∙Cp}_{to}\right)}_{s}\frac{{dT}_{s}}{dt}={\dot{Q}}_{col}-{\dot{Q}}_{ORC}-{\left(UA\right)}_{s}∙\left(T_{s}-T_{a}\right)$$

where $m_{to}$ and $C_{p,to}$ are the mass and heat capacity of the thermal oil, ${\dot{Q}}_{col}$ is the amount of heat absorbed in the collector given by equation (22), ${\dot{Q}}_{ORC}$ is the heat load of the ORC, ${\left(UA\right)}_{s}$ is the product of the area of the thermal storage tank and the overall heat loss coefficient whose value was 11.1 W/K [28], $T_{s}$ is the initial temperature of the tank, and $T_{a}$ is the room temperature. Equation (28) can be written as shown in equation (29):

(29) $$T_{s,new}=T_{s,i}+\frac{\Delta t}{{{\dot{m}}_{to}∙Cp}_{to}}\left[{\dot{Q}}_{col}-{\dot{Q}}_{ORC}-{\left(UA\right)}_{s}∙\left(T_{s}-T_{a}\right)\right]$$

where $T_{s,new}$ is the fluid temperature in the thermal storage tank by taking an hourly step.

### 2.4 Economic analysis

In the economic analysis of thermal systems, the total cost of production must be calculated ($C_{TP}$), which depends on the total investment cost ($C_{TI}$) and the maintenance and operation cost ($C_{MF}$) given by equation (30) [37].

(30) $$C_{TP}=C_{TI}+C_{MF}$$

where the total investment cost $\left(C_{TI}\right)$ is calculated by the mean of equation (31).

(31) $$C_{TI}=I_{FA}+\xi$$

where $I_{FA}$ represents the system's fixed-asset investment, which is the sum of the direct costs $\left(C_{D}\right)$ and the indirect costs $\left(C_{I}\right)$ given by equation (32).

(32) $$I_{FA}=C_{I}+C_{D}$$

In addition, ξ represents other costs which include the start-up costs $\left(C_{A}\right)$, the initial working capital cost ($C_{CIW}$), the research and development costs ($C_{DI})$, and the funding costs during the construction phase $\left(C_{CF}\right)$, according to equation (33).

(33) $$\xi =C_{A}+C_{CIW}+C_{DI}\;{+C}_{CF}$$

On the other hand, there are direct costs, which include the purchase equipment cost of the system, the piping, installation, and assembly costs, the instrumentation and control cost, the electrical equipment, civil works, and work area costs. Table 2 shows the equations used to calculate the purchase equipment cost of each equipment of the system, which are a function of area and power rate.

**Table 2:** Equations used in the economic model.

Component	Cost Equation	Reference
**ITC1**	$Z_{ITC1}=10000+342\;{{∙(A}_{ITC1})}^{0.91}$	[38,39]
**ITC2**	$Z_{ITC2}=10000+342\;{{∙(A}_{ITC2})}^{0.91}$	[38,39]
**ITC3**	$Z_{ITC3}=10000+342\;{{∙(A}_{ITC3})}^{0.91}$	[38,39]
**T1**	${Z_{T1=}10}^{\left(\theta_{T1}\right)}$ $\theta_{T1}=2.70+1.44+\log\left(Wt\right)-0.178∙{\left[\log\left(W_{T}\right)\right]}^{2}$	[39,40]
**P1**	${Z_{P1=}10}^{\left(\theta_{P1}\right)}$ $\theta_{P1}=3.39+0.054+\log\left(W_{P1}\right)-0.154∙{\left[\log\left(W_{P1}\right)\right]}^{2}$	[39,40]
**P2**	${Z_{P2=}10}^{\left(\theta_{P2}\right)}$ $\theta_{T2}=3.39+0.054+\log\left(W_{P2}\right)-0.154∙{\left[\log\left(W_{P2}\right)\right]}^{2}$	[39,40]
**P3**	${Z_{P3=}10}^{\left(\theta_{P3}\right)}$ $\theta_{p3}=3.39+0.054+\log\left(W_{P3}\right)-0.154∙{\left[\log\left(W_{P3}\right)\right]}^{2}$	[39,40]
**Collector Solar (CS)**	$Z_{Collector}=\left(150+90\right){∙A}_{Collector}$	[41]
**Thermal storage tank (TK)**	$Z_{TK}=35\;USD/A_{Collector}$	[41]

From the total purchase equipment cost of the system, the direct costs of pipe and fittings cost were considered at 9% [42], the installation and assembly cost at 20% [42], the instrumentation and control cost at 5% [43], electrical equipment and materials cost in 5% [42], the civilian work in 5% [42], and the total work area cost in 10% [42].

Similarly, the indirect costs were taken from the total assets of the project, which are the engineering cost at 30% [42], construction and contingencies cost at 15% [42], as well as other costs, such as the related to start-up in 10% [43], the labor capital in 10% [42], and the provision during the construction phase in 15% [42]. Finally, the operation and maintenance costs associated with supervision at 15% [44], parafiscal expenses cost at 35% [42], the maintenance cost at 6% [44], and the miscellaneous costs at 15% [44] were also estimated for total project assets.

To obtain the levelized cost values, the constant scaling factor is defined (CELF), which relates the costs of the project's first year to an equivalent annual annuity, according to equation (34).

(34) $$CELF=CRF∙\frac{\lambda \left(1-\lambda^{n}\right)}{1-\lambda }$$

where n is the year number of the project, and λ is defined by equation (35).

(35) $$\lambda =\frac{1+r_{n}}{1+i_{eff}}$$

where $r_{n}$ is the nominal reason for escalation, and the CRF is defined by equation (36).

(36) $$CRF=\frac{i_{eff}∙{\left(1+i_{eff}\right)}^{n}}{{\left(1+i_{eff}\right)}^{n}-1}$$

where $i_{eff}$ is the reason for interest. Table 3 shows the values used in the economic model.

**Table 3:** Constants used in the economic model.

Economic constants	Value	Ref
Interest rate, $i_{eff}$	10%	[45,46]
Maintenance factor, $m_{f}$	8%	
Nominal scaling ratio, $r_{n}$	5%	[40,47]
Plant lifetime, n	20 year	[40,48]
Working hours, τ	7446 h	[49]

#### 2.4.1 Economic indicators

The LCOE allows the estimation of the sale price of energy to meet the investment costs of the project, was estimated by equation (37) [50].

(37) $$LCOE=\frac{\sum_{n=0}^{N}\left(C_{n}+FE_{n}+O\&M_{n}\right)}{\sum_{n=0}^{N}\frac{E_{n}}{{\left(r+1\right)}^{n}}}$$

where $C_{n}$ is the cost of each component, n is life cycle time in years, O&M is the cost to operate and maintain the process, $E_{n}$ is the energy produced annually, $FE_{n}$ is the employed fuel costs, and r is the discount rate.

The specific cost of the investment (SIC), is given by equation (38) [51].

(38) $${SIC}_{Solar-ORC}=\frac{C_{Solar-ORC}}{{\dot{W}}_{net}}$$

where $C_{Solar-ORC}$ is the total cost of the renewable system, and ${\dot{W}}_{net}$ is the net power produced.

The payback period (PBP) was expressed according to equation (39) [52].

(39) $$PPBP=\frac{C_{AP}}{\left(1-t\right)∙\left(S_{annual}-C_{TD}\right)+C_{AD}}$$

where $C_{TD}$ is the capital depreciation, $C_{AP}$ is the annual production cost, $S_{annual}$ is the annual sales revenues direct costs, ${t\;is\;the\;sum\;of\;federal\;and\;state\;income\;tax\;rateC}_{AD}$ is the annual depreciation, and.

Finally, to evaluate the performance between conventional systems and ORC, when providing electrical charge, the Relative Annual Benefit (RAB) [25] was considered, according to equation (40).

(40) $$RAB={TAC}_{con}-{TAC}_{ORC}$$

where ${TAC}_{con}$ is the annual purchase cost of energy from the electrical network operator in USD/year at the cost of 543 COP/kWh, estimated using equation (41).

(41) $${TAC}_{con}=\sum_{i=1}^{N}\left({E_{c}∙P}_{b}\right)∙S_{s}$$

where $E_{c}$ is the electrical energy demand of the load in kWh, $P_{b}$ is the cost of buying energy from the network operator in kilowatts per hour, and $S_{s}$ is the step size of load variation in the studied period (1 h).

The annual cost of the solar-ORC system includes the purchase equipment costs, the maintenance, and operating costs, the purchase and sale of electricity cost, and it is estimated according to equation (42).

(42) $${TAC}_{ORC}={\left[\frac{i_{eff}}{1-{\left(1+i_{eff}\right)}^{n}}\right]{∙m}_{f}∙C}_{inv}\sum_{i=a}^{N}\left({E_{b}∙P}_{b}\right)∙S_{s}-\sum_{i=a}^{N}\left({E_{s}∙P}_{s}\right)∙S_{s}$$

where $E_{b}$ is the kilowatts per hour of energy purchased from the grid, $E_{s}$ is the energy sold or delivered to the grid, $P_{s}$ is the selling price of energy, $C_{inv}$ is the purchase equipment costs of the solar-ORC, which were estimated according to the equations shown in Table 2. Also, the economic constants $m_{f}$ , $i_{eff}$ , and n were defined in Table 3.

Equation (40) allows to compare in economic terms the performance of both configurations. A high value of RAB indicates that the system ORC is more viable than the traditional system. From equation (42), the sale of energy ($E_{b}$) is zero when the power demand of the load exceeds the maximum generation capacity of the ORC, which requires an operational scenario of buying energy to balance the system. On the other hand, when the energy demand of the load is lower than that generated, the additional energy is sold to the network.

### 2.5 Life cycle assessment of the Solar-ORC system

**Fig. 4:**
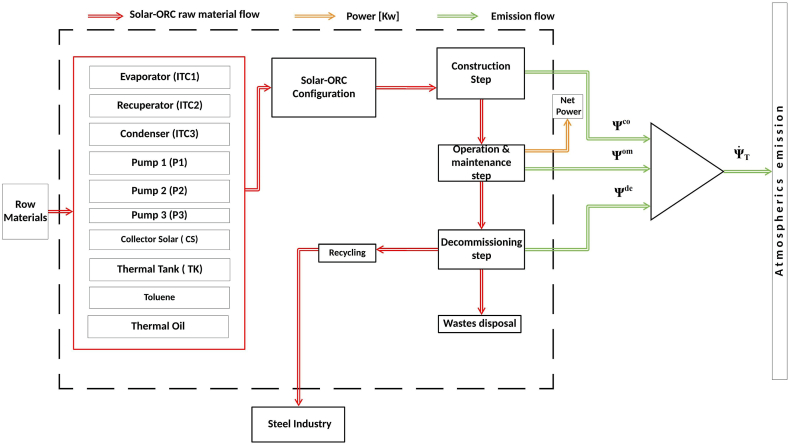
Life Cycle boundary of the Solar-ORC.

The Life Cycle Assessment of the solar-ORC system was selected as the approach to quantify the potential environmental impact, to obtain a sustainable solution. Fig. 4 shows the LCA boundary considered in the study. The life-time considered for the LCA was twenty years, and the raw materials for the solar-ORC devices were considered in the construction phase, some components such as the valves and pipe were not considered. The Eco-indicator 99 method was proposed to determine the organic working fluid and thermal oil potential environmental impact.

The study was conducted considering the potential environmental impact of each phase in the thermal system life-cycle, which are the construction, the commissioning, and the decommissioning phase. Finally, the environmental impact of the complete system is determined for the two solar-ORC configurations understudy, as shown in Fig. 4.

The potential environmental impact of the power components of the system can be calculated according to equation (43)

(43) $$\psi_{i}=\varphi_{i}∙\alpha_{i}∙W_{i}$$

where $\varphi_{i}$ is the Eco 99 coefficient of the components in 86 mPts/kg considering stell, $\alpha_{i}$ is related to the steel type of the pump (14 kg/kWh) and the turbine (31.22 kg/kWh) [53], and $W_{i}$ is the power of the equipment. For the heat exchangers, the potential environmental impact can be determined by equation (44).

(44) $$\psi_{i}=\varphi_{i}∙\rho ∙A_{i}∙\delta$$

where ρ is the density of steel ($7930\;kg/m^{3}$), $A_{i}$ is the heat transfer area of the equipment, and δ is the thickness of the material 0.002 m [53].

The Eco-indicator 99 coefficient for the solar collector was taking into account the material by which it is constituted with its respective Impact, as shown in Table 4.

**Table 4:** Eco-indicator 99 for 1 m^2^ of flat-plate collector solar [54].

Material	Amount	Eco 99 [ Pt/m^2^]
Copper	3 kg	8.1
Aluminum (Collector frame)	3.9 kg	4.0
Soft solder	0.059 kg	1.3
Solar glass	9.1 kg	1.0
Sheet Rolling	2.8 kg	0.5
Transport lorry	28.4 tkm	0.4
Rock Wool	2.4 kg	0.5
Propylene glycol	0.88 kg	0.3
Synthetic rubber	0.76 kg	0.3
rest		0.8
Total		17.2

The Eco99 for the thermal storage tank was taken from 104 Pt/m^3^ [54]. Therefore, the potential environmental impact of a device is calculated with equation (45).

(45) $$\psi_{i}^{LCA}=\psi_{i}^{co}+\psi_{i}^{om}+\psi_{i}^{de}$$

and the total environmental impact of the thermal system can be calculated according equation (46).

(46) $${\dot{\Psi }}_{T}=\sum_{i=1}^{kth}\psi_{i}^{LCA}/\left(\tau ∙n\right)$$

On the other hand, the environmental Impact of the toluene as a working fluid (toluene) and thermal oil (73.5% Diphenyl Oxide) were considered in the three stages of the life-cycle. Also, in the ORC, the annual fluid losses in the operation stage were set from 10% [53] and 3% for the decommissioning stage. The environmental impact coefficients used for the LCA calculation were available in the Eco-indicator 99 database [55].

### 2.6 Particle Swarm Optimization method

The Particle Swarm Optimization method is an evolutionary search technique well-known in engineering optimization problems [56], which has been implemented in many areas of knowledge. In this algorithm, the particles of the swarm travel through a search space to find a potential solution, and each member learns from the best performer of all. The PSO consists of several steps; in the first, the positions and speeds are randomly initialized. Then, each particle is evaluated in the target function; in the next iteration, the values of the best global and local positions are updated. Finally, the particle velocity is updated for the new iteration according to equation (47).

(47) $$v_{i}^{k+1}=wv_{i}^{k}+c_{1}r_{1}\;\left(p_{i}^{k}-x_{i}^{k}\right)+c_{2}\;r_{2}\;\left(p_{j}^{k}-x_{i}^{k}\right)$$

where the term $v_{i}^{k+1}$ corresponds to the new speed of each particle, w is the inertia factor, $r_{1}$ and $r_{2}$ are random numbers between zero and one, $c_{1}$ and $c_{2}$ are the coefficients of acceleration.

**Fig. 5:**
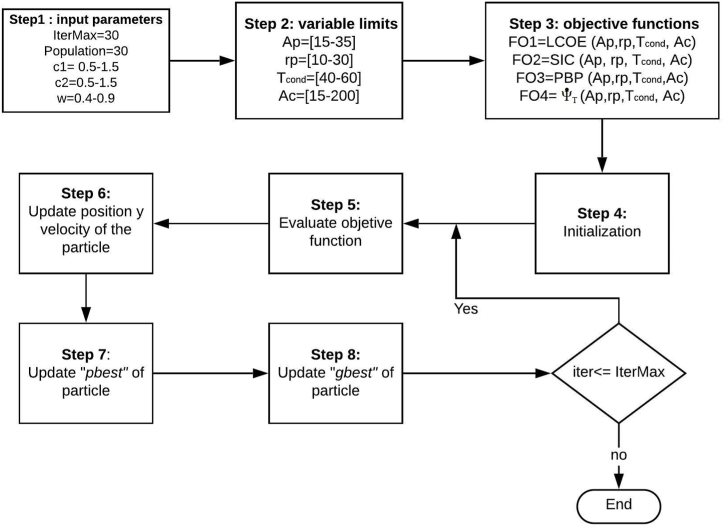
Flow chart of the PSO algorithm.

The position of each particle in the iteration k + 1 is calculated by equation (48).

(48) $$x_{i}^{k+1}=x_{i}^{k}+v_{i}^{k}$$

where $x_{i}^{k}$ and $v_{i}^{k}$ are position and speed in iteration k-1. Fig. 5 shows the step-by-step flow chart for the PSO implementation, along with the values of the constants considered for the search process.

## 3 Results and discussion

The ORC was powered by a medium temperature flat-plate solar collector to supply the energy demand of a building. In this study, two solar-ORC configurations were evaluated, the simple organic Rankine cycle and the recuperative organic Rankine cycle under a measured solar field radiation data from a geographical area of Colombia called Las Flores one field with the most solar potentials in the country. The energy, exergy, thermo-economic and comparative environmental study of the thermal systems were carried out, and later the evolutionary optimization results are presented, which allowed determining the proper values of the decision variables to optimized the performance indicators considered, obtaining a sustainable renewable thermal system solution feasible to be implemented. The main values used for the simulation of the different configurations of the solar-ORC are presented in Table 5.

**Table 5:** Main values used in the configurations.

Configuration	Parameters	Value	Units
SORC/RORC	Turbine isentropic efficiency	85	%
SORC/RORC	Pump isentropic efficiency	85	%
SORC/RORC	Cooling water temperature	25	°C
SORC/RORC	Pinch Point condenser temperature	10	°C
SORC/RORC	Pinch Point evaporators temperature	30	°C
SORC/RORC	Condensation temperature	40	°C
SORC/RORC	Pressure ratio (P1, P2)	2.5	–
SORC/RORC	Pressure ratio (P3)	20	–
RORC	Recuperator efficiency	0.85	–

Table 6 shows the main thermodynamic and physical properties obtained for the RORC, which were obtained based on the data presented in Table 5 and estimated for a typical day of March measured in Las Flores station. The thermodynamic properties of the solar-SORC system are shown in Table A1.

**Table 6:** Thermodynamics Properties Solar-RORC configuration.

Point	T [K]	P [kPa]	h [kJ/kg]	s [kJ/kg-K]	s- $\left(s-s_{0}\right)$ [kJ/kg-K]	$\dot{E}$ [kW]	$\dot{m}$ [kg/s]
**1**	313.15	7.89	-132.35	-0.38	0.08	0.24	0.457
**2**	313.20	157.85	-132.15	-0.38	0.08	0.32	0.457
**2r**	382.79	157.85	-1.87	-0.01	0.46	8.80	0.457
**2rls**	399.96	157.16	33.02	0.08	0.55	12.60	0.457
**2rvs**	399.96	123.54	370.36	0.95	1.41	49.12	0.457
**3**	484.75	89.92	526.76	1.28	1.75	74.82	0.457
**4**	422.74	7.89	424.98	1.33	1.79	22.39	0.457
**4r**	331.92	7.89	294.70	0.98	1.45	9.89	0.457
**4rvs**	313.15	7.89	271.65	0.91	1.37	9.09	0.457
**5**	514.75	101.23	488.48	–	1.28	41.95	1.000
**5a**	514.81	202.46	488.62	–	1.28	41.97	1.000
**5avs**	484.27	199.36	418.63	–	1.08	30.98	1.000
**5ls**	410.13	196.42	262.98	–	0.63	11.38	1.000
**6**	402.66	193.58	248.35	–	0.58	10.18	1.000
**7**	298.15	101.30	104.92	0.37	0.00	0.00	8.841
**7vs**	303.15	87.48	125.82	0.44	0.44	1.53	8.841
**8**	303.44	73.44	127.02	0.44	0.44	1.71	8.841
**9**	453.15	101.23	350.90	–	0.89	21.80	1.000
**9a**	453.22	202.46	350.97	–	0.88	21.80	1.000
**10**	467.08	101.23	380.83	–	0.97	25.65	1.000

Table 7 shows the results obtained for the main study parameters considered for each of the configurations. These values were obtained with the base conditions reported in Table 3 at the time of the greatest solar radiation (12:00 p.m.).

**Table 7:** Values of the main output parameters of the system in its base condition.

Zone	Parameters	Unit	SORC	RORC
**Las Flores**	Source Temperature, $T_{5a}$	°C	250	250
	Solar irradiation, $I_{0}$	${W/m}^{2}$	914	914
	Heat useful gain, ${\dot{Q}}_{Col}$	kW	48.78	48.78
	Net Power Output, ${\dot{W}}_{net}$	kW	46.80	49.29
	Thermal efficiency ORC*,*$\eta_{I\;ORC}$	%	13.92	19.31
	Levelized cost of energy, LCOE	USD/kWh	0.3668	0.4008
	Specific Investment Cost, SIC	USD/kWh	2821.43	3083.1
	Environmental Impact, ${\dot{\Psi }}_{T}$	mPts/kWh	15479.82	15498.83
	Payback Period, PBP	year	11.68	12.77

For the source temperature of 250 °C, the solar-RORC system presents an increase of 5.32% in the power output with respect to the solar- SORC system. Also, the rate of heat production in this radiation condition is 48.70 kW. However, in economic terms, the solar-SORC system presents more favorable results in contrast to the configuration RORC as a consequence of the reduced number of devices on the configuration, which implies a lower purchase of equipment cost.

**Fig. 6:**
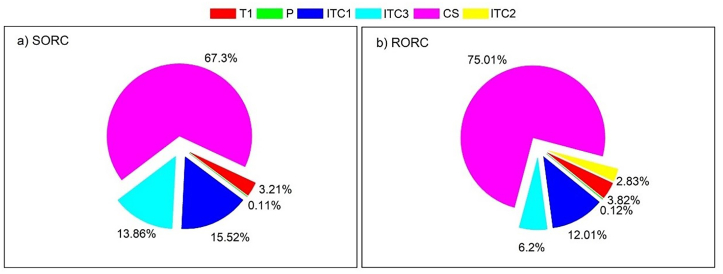
Exergy destruction distribution in the system.

Fig. 6a and b show the percentage of exergy destruction that the components of the configurations have. It can be seen that the collector is the equipment that has the highest rate for SORC (67.3%) and RORC (75.01%), which makes it the component with the greatest potential for improvement from the exergetic point of view. Next, there is the heat transfer equipment which represents 29.35% of the SORC and 21.04% of the RORC. Then there is the turbine of the SORC (3.21%) and RORC (3.82%). Finally, the pumps were totaled and represented less than 1% in the systems.

Table 8 shows the results of the environmental impact assessed under the base conditions for the RORC. The total impact of the process is 15498.3 mPts/kWh. The strong impact that the system has is due to the contribution that the thermal fluid makes to the system. It can be seen that the total quantity of thermal oil was 50.5 ton in the construction phase, which is associated with its high value of Eco-99 makes a great influence to the overall Impact [57]. Next is the turbine and exchange equipment (ITC1, ITC2, and ITC3) of heat. However, the contribution of these components compared to oil is very low. The results for the solar-SORC are shown in Table A2.

**Table 8:** LCA results for the solar-RORC.

Component	Material	Eco-99 [mPts/kg]	Quality [kg]	Y^co^ [mPts]	Y^om^ [mPts]	Y^de^ [mPts]	Y [mPts]
ITC1	Stainless Steel	86	128.78	11629	0	554	12183
ITC2	Stainless Steel	86	264.39	23874	0	1137	25011
ITC3	Stainless Steel	86	50.12	4526	0	216	4741
TK	Stainless Steel	0.1040^b^	50.00^c^	5.5	0	0.260	6
SC	mixture^a^	0.0172^b^	200.00^c^	3.6	0	0.172	4
T1	Stainless Steel	86	1515.42	136842	0	6516	143359
P1	Stainless Steel	86	1.77	160	0	7.6	167
P2	Stainless Steel	86	1.96	177	0	8.4	185
P3	Stainless Steel	86	1.37	124	0	5.9	129.92
Thermal Oil	73.5% Diphenyl Oxide	46467	50500.00	2409941255	240994125	63357755	2714293134
Fluid	Cyclohexane	2639	270.37	732737	73274	19264	825275

a Table 4 shows the main materials of the flat-plate collector solar.

b For the tank (TK) the Eco99 in units of mPts/m^3^; while for the collector solar (CS) at mPts/m.^2^.

c For the thermal storage tank (TK) the units are in m^3^ and m^2^for the collector solar (CS).

### 3.1 Daily radiation simulation

**Fig. 7:**
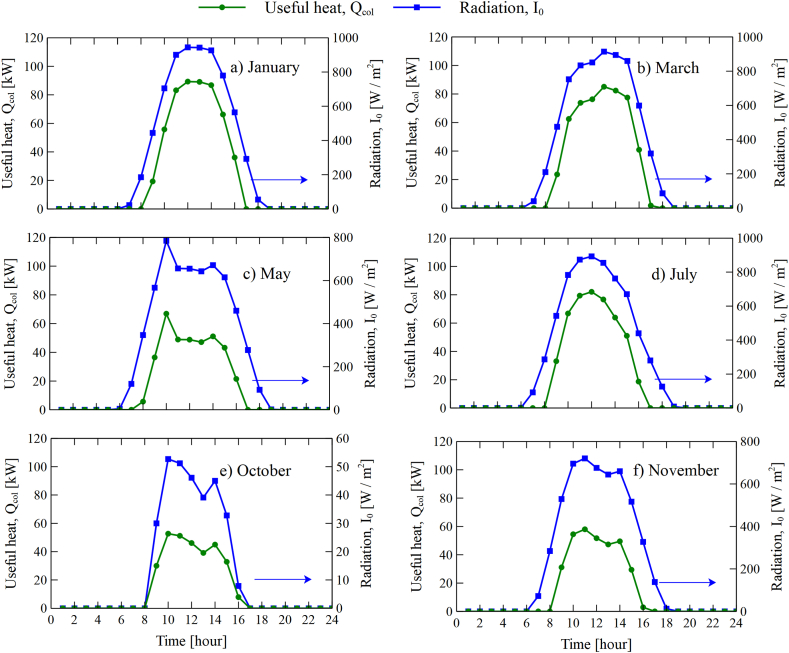
Collector heat variation and solar radiation during a simulation day: (a) January, (b) March, (c) May, (d) July, (e) October, and (f) November.

Fig. 7 shows the heat gain profiles of the collector and radiation for six months representative of the year. The results show an increase in heat as the radiation increases, peaking at midday and then decrease as the radiation declines.

Additionally, it is observed that are there two peaks of thermal radiation in some months in Fig. 7. The peaks of thermal radiation found in Fig. 7c and e are due to atmospheric transmittance. In our case, the atmospheric transmissivity values in our model change from month to month. Additionally, weather phenomena such as cloudiness could have affected the values during the modeling.

**Fig. 8:**
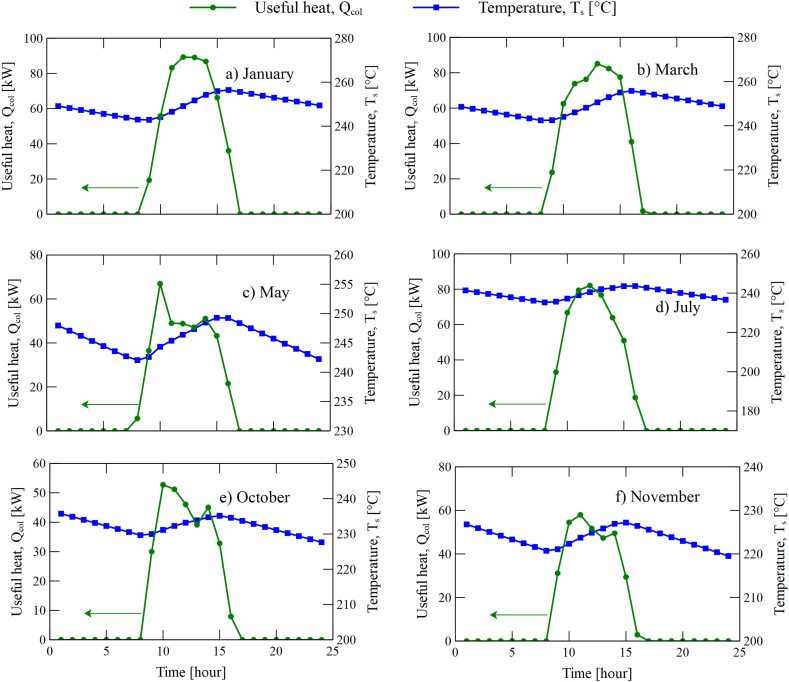
Hourly variation of tank outlet temperature: (a) January, (b) March, (c) May, (d) July, (e) October and (f) November.

On the other hand, based on the maximum radiation values obtained in each zone, the temperature variation in the transitory state inside the tank during each hour of the day was determined. Fig. 8 shows a reduction in the tank outlet temperature. This reduction arises from the constant load that the tank need to supply to the ORC to guarantee the system's operability in hours without radiation. From 9:00 a.m., the flow of thermal oil starts to heat when it passes through the solar collector. This heating generates a heat contribution to the thermal tank that increases its temperature, as observed in the area inside the dome, as in Fig. 8. Finally, when there is no solar radiation, the system temperature tends to decrease gradually. This same behavior is observed in each of the day's representatives of the months considered in the study, according to Fig. 8.

### 3.2 Hourly simulation of the sale, purchase, generation, and consumption of electrical energy

**Fig. 9:**
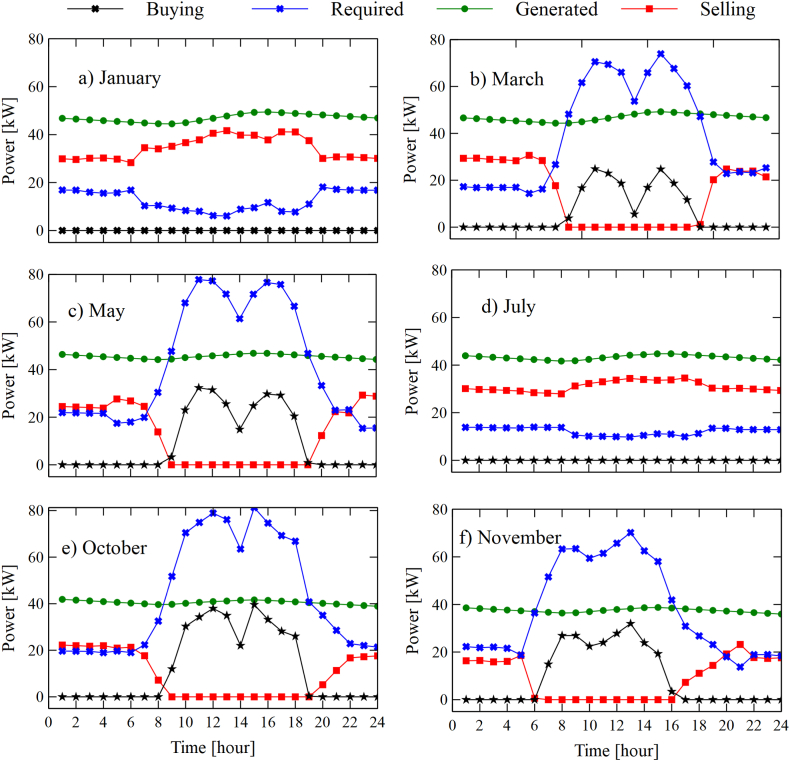
Variations in consumption, generation, sale, and purchase of energy for six representative months, configuration SORC: (a) January, (b) March, (c) May, (d) July, (e) October, and (f) November.

In this section, a study of the energy performance of the integrated system is carried out building-ORC. The energy consumption data was taken from a record of hourly electricity consumption in a building at the Universidad del Atlántico, which was acquired through network analyzers at different points. Subsequently, the costs of selling and buying energy were determined, which are regulated by the Energy and Gas Regulation Commission (CREG) in Colombia. The study considers all the days of the month to have the same behavior in terms of radiation. Furthermore, the system was simulated by continuously considering a typical day of each month. Also, six months have been selected for simplicity when showing the results (see Fig. 9). Fig. 9 shows that in January (Fig. 9a.) and July (Fig. 9d), there is no increase in the energy demand required by the system during working hours. This is because during these months, the University is on vacation, and therefore there is not a high demand for electricity, which usually comes from classrooms and laboratories. In this scenario, in January and July, there will always be a sale of energy delivered to the national interconnection system at 0.0673 USD/kWh, while the energy purchase will be zero.

The production of electricity for January (47 kWh) is higher than in July (35.55 kWh), which represents a decrease of 24.36%. One explanation for this behavior is due to the high levels of radiation in January concerning those of July, which impacts an increase in the useful heat gain in the collector and, therefore, an increase in the temperature of the tank that feeds the system SORC. Therefore, a significant reduction in the tank outlet temperature can be expected for July compared to the variations that occur in January. Also, it must be taken into account that the model was simulated continuously to have a real approximation in terms of temperature variation in time.

It is pertinent to emphasize that the production of energy at almost constant levels at night is due to the high thermal capacitance of the tank, which allows for maintaining the entrance temperature to the ORC system without showing strong decay in hours in the absence of radiation.

On the other hand, for the rest of the months, they exhibit similar behavior. In the case of May, it is observed that in the early morning hours, the system's power generation is higher than the load demand. Therefore, there is an energy sale from 12:00 a.m. to 7:00 a.m. From this time onwards, the demand for the load increases significantly due to the start of classes and administrative activities in the building, and consequently, the system SORC cannot meet the entire demand, and energy purchases must be made to compensate. Finally, after 10:00 p.m., power is delivered to the interconnection system. This same behavior is evident for March, October, and November, as shown in Fig. 9.

**Fig. 10:**
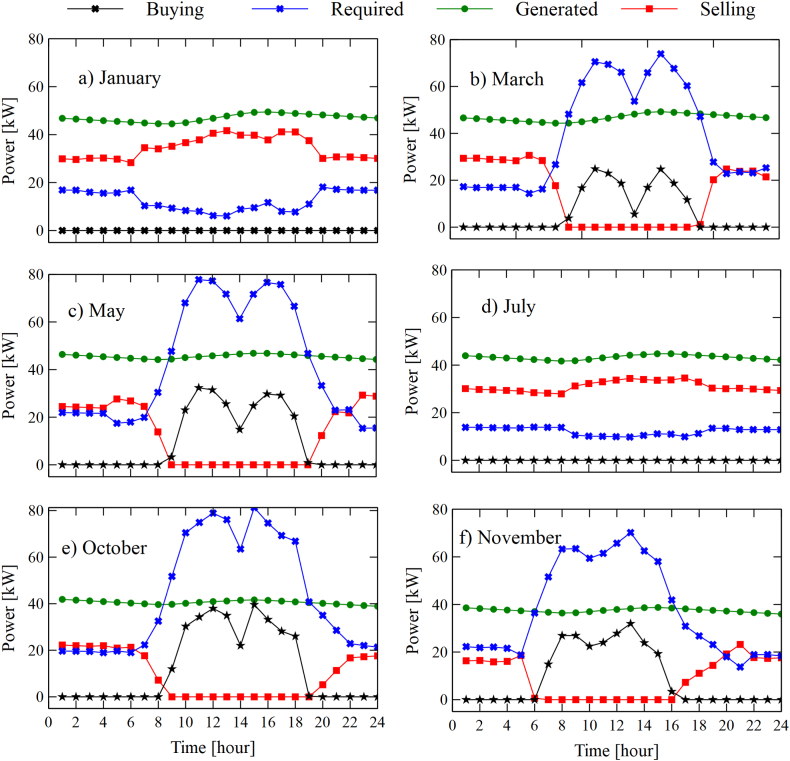
Variations in consumption, generation, sale, and purchase of energy for six representative months, configuration RORC: (a) January, (b) March, (c) May, (d) July, (e) October, and (f) November.

Fig. 10a and b, Fig. 10c, d, Fig. 10e, and Fig. 10f show the variation in the generation, consumption, purchase, and sale of electrical energy of the integrated system building -RORC. The behavior found is similar to that found by the SORC.

However, the system RORC has an advantage over the system SORC. Taking as a reference the month of May, the generation of electrical energy of the SORC (45.51 kWh) is less than the system RORC (47.86 kWh), which represents an increase of 5%. Therefore, in terms of power generation up to this point, the system RORC presents greater viability as it reduces the number of kWh purchased from the network operator in hours when demand exceeds generation. However, it is necessary to study from an economic point of view to determine how profitable it is to implement these systems, which will be studied in the following sections of this document.

**Fig. 11:**
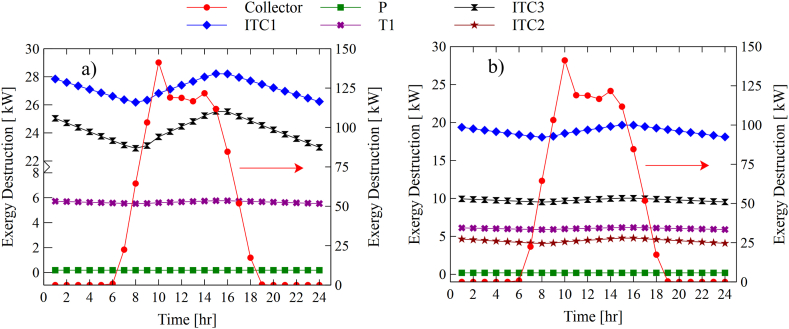
Hourly destroyed exergy of the components, a) SORC, b) RORC.

Fig. 11a and b show the hourly behavior of the destroyed exergies of the main components of the configurations. The typical day of May was taken to study its variations. It is observed that the solar collector is the equipment that presents the highest rate of exergy destroyed in both configurations, with a maximum value of 141.24 kW. These peak values are due to the incident radiation on the collector surface (see Fig. 7c). The energy destruction rate increases as the radiation to the solar collector increases. However, the ORC cycle components tend to experience a slight decrease due to a reduction in the source temperature. Then it increases at peak hours and finally decreases. It is also observed that the exergy destroyed in the components of the RORC configuration is lower in contrast to the SORC configuration. This is due to the effect of the heat recuperator that allows it to take advantage of a part of the internal heat of the system and, thus, reduces the demands from the thermal source and, consequently, better use of the useful heat of the system.

### 3.3 Economic analysis of the system

**Fig. 12:**
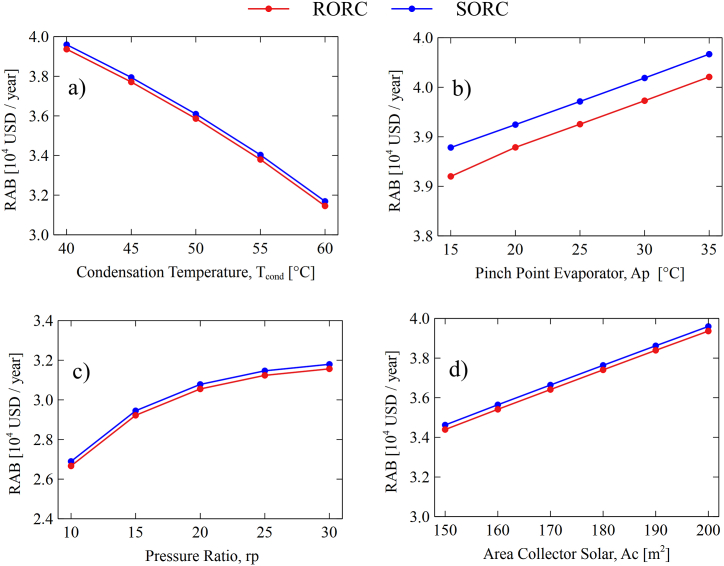
Variation of the relative annual cost for different system variables SORC and RORC: (a) Condensation temperature, (b) Pinch point evaporator, (c) Pressure ratio, and (d) Area collector solar.

In this section, a study is made of the economic performance of the different parameters considered in this study. To have a practical evaluation between the performance of traditional systems and ORC, the definition has been made RAB (Relative Annual Benefit). The results are shown in Fig. 12a, Fig. 12b, c, and Fig. 12d.

Fig. 12a shows that increasing the system's condensing temperature decreases the RAB for the SORC (19.96%) and RORC (20.08%). Therefore, the increase in the condensation temperature of the system tends to have an unfavorable effect in terms of the relative annual cost (RAB). In this scenario, the increase in the condensation temperature tends to lower the acquisition costs of heat transfer equipment since these are a function of the heat exchange areas. The higher the condensing temperature, the smaller the area required for this equipment and, therefore the lower its acquisition cost. However, the influence of the investment costs of the equipment is not significant concerning the influence that the system has in terms of the reduction of electrical energy due to the increase of the condensation temperature. The effect of the increase in the condensing temperature has an impact on a significant reduction in the power generated in the turbine. This is basically due to a decrease in the useful work of the current entering the turbine, which implies a lower power generation capacity. In this sense, decreasing the condensation temperature of the systems implies economic disadvantages, prevailing the associated cost for energy reduction in contrast to the investment costs of the system's equipment.

Fig. 12b shows that the increase in a pinch of the evaporator reduces the RAB systemic SORC (2.40%) and RORC (2.60%). This behavior is related to the minimum difference in the temperature approach of the thermal fluid and the working fluid. The smaller this difference, the greater the power delivered by the turbine. However, reducing the pinch of the evaporator implies a greater area and, as a result, an increase in the cost of acquisition of the equipment. However, the increase in energy due to the reduction of the pinch of the evaporator compensates for the increased investment cost so that the RAB in both configurations is satisfactorily favored by the increase of this variable.

Similar behavior was found for the pressure ratio increase shown in Fig. 12c in which the RAB del system SORC (18.22%) and RORC (18.38%) increased. The pressure ratio is one of the parameters that most Impact the production of energy since a higher-pressure ratio implies a higher evaporation pressure and, therefore, more electricity generation. Finally, the collector area also increases the RAB systemic SORC (14.45%) and RORC (14.37%) since a larger area rises the amount of useful heat gain in the collector and, consequently, a contribution to the increase in the storage tank outlet temperature, i.e., an increase in the temperature of the heat source to the system ORC.

The RAB is a parameter that allows determining both configurations' performance in economic terms. A high RAB value indicates that the ORC system is more viable than a conventional power system. This is because the total annual costs (${TAC}_{ORC}$), which include operating costs, maintenance, etc., are lower than the total annual costs of a conventional system (${TAC}_{Con}$). In that sense, Fig. 12 shows in all the cases that the RAB of the SORC is higher than that of the RORC. This is because the SORC does not have a heat recovery unit, which causes the investment cost to be lower with respect to the RORC. In addition, maintenance costs are also reduced. Therefore, the operating cost of the SORC system will be lower for the RORC, even though the RORC has a higher energy production. The energy production of the RORC is not significantly high compared to the SORC. Consequently, maintenance and power purchase costs (when demand exceeds generation) prevail over power generation.

**Fig. 13:**
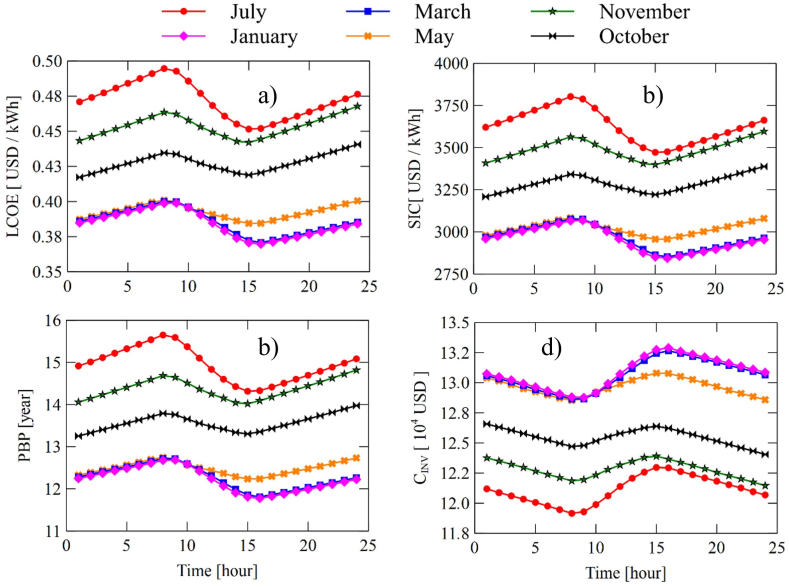
Hourly behavior of system economic variables SORC: (a) LCOE, (b) SIC, (c) PBP, and (d) $C_{INV}$.

In addition, Fig. 13 displays the hourly variations of the economic indicators of the system. This analysis seeks to find the hours of greatest thermo-economic viability of the system, that is, to see the optimal points during the day. In general terms, these indicators have very similar behavior during the day. In hours below 8:00 a.m., there is an increase in the LCOE (Fig. 13a), SIC (Fig. 13b), and PBP (Fig. 13c). This behavior is because, in the first hours of the day, there is a reduction in the temperature of the source (Fig. 7) product of the continuous load that the tank is yielding to the system ORC. This implies that the indicators LCOE, SIC, and PBB increase because of the lower energy generation of the system. Then, in hours when there is radiation, the behavior of the indicators is to decrease due to an rise in the source temperature and, therefore, a slight increase in power generation. Finally, in hours when there is no radiation, the system decreases its economic indicators again. Fig. 13 also shows the maximum and minimum limits presented by these indicators. For these months of study, July is the month with the highest values in the indicators LCOE (0.4946 USD/kWh), SIC (3802 USD/kWh), and PBP (15.6 years); while January's lowest in LCOE (0.3990 USD/kWh), SIC (3068 USD/kWh), and PBP (12.6 years) all read at 8:00 a.m.

On the other hand, the behavior observed by the investment cost of the equipment, shown in Fig. 13d, is opposite to that found in the indicators previously studied. The explanation for this trend lies mainly in the areas for the case of heat and power transfer equipment for pumps and turbines. The lower the temperature at which the working fluid enters from the thermal source, the smaller the area of exchange required and, therefore, the lower the acquisition cost of the equipment; a similar case occurs for the pumps and turbines. In this sense, in hours with no radiation when the entry temperature to the SORC system tends to decrease, there will be a decrease in the investment cost; while in hours where there are radiation and the source temperature increases, there will be an increase in the acquisition cost of the equipment as shown in Fig. 10d.

**Fig. 14:**
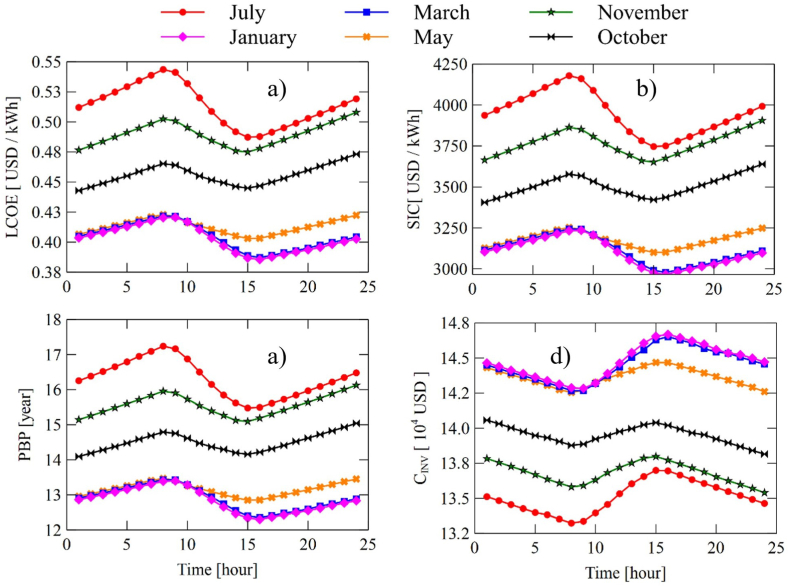
Hourly behavior of system economic variables RORC: (a) LCOE, (b) SIC, (c) PBP, and (d) $C_{INV}$.

The behavior found for the system RORC shown in Fig. 14, is similar to the SORC. However, the system RORC presents a slight increase in the investment cost due to the incorporation of the heat recuperator into the system, which in turn impacts a slight increase in the indicators LCOE, SIC, and PBP.

For the July system, it is the one with the highest values in the LCOE (0.5436 USD/kWh), SIC (4179 USD/kWh), and PBP (17 years); while January's lowest in LCOE (0.4206 USD/kWh), SIC (3205 USD/kWh), and PBP (13.4 years) all read at 8:00 a.m.

In general terms, it was found that the RORC system presents greater viability in thermal power generation since it presents a higher production rate concerning the SORC configuration, which was evidenced in Fig. 9, Fig. 10. Also, the results found as a result of the analysis of the relative annual cost (RAB), shown in Fig. 12, revealed that the SORC configuration showed better viability concerning the RORC system. Although this indicator only took into account the initial investment cost of the equipment, it offered a rough estimate of the systems' behavior, which was demonstrated in the hourly study of the economic performance of the configurations shown in Fig. 13, Fig. 14, in which the RORC configuration presented higher values with respect to the SORC system.

### 3.4 Thermoeconomic and environmental optimization

In this section, the settings are optimized SORC and RORC to determine the optimal values of the decision variables selected for this study. These results are aimed at providing a realistic estimate of the costs associated with the application of these configurations in buildings. Also, an indicator is included that measures the environmental impact of these systems. In the first instance, the optimization process was carried out considering a radiation value in a specific month of the year. Since performing optimizations, considering hourly variations implies a high computational cost. For this reason, hourly studies of the behavior of the economic variables were carried out to have a future projection of these variations and evaluate the impact they have. The month of May was considered to carry out the optimization since it was the month that presented the greatest accumulated radiation. Subsequently, the algorithm PSO, is an easy-to-implement search algorithm, with the objective functions defined in equations (49), (50), (51), (52)).

(49) FO1=LCOE(Ap,Tcond,rp,Ac)

(50) FO2=SIC(Ap,Tcond,rp,Ac)

(51) FO3=PBP(Ap,Tcond,rp,Ac)

(52) $$FO4={\dot{\Psi }}_{T}\left(Ap,Tcond,rp,Ac\right)$$

Subsequently, the search range of the decision variables considered in the study was defined, shown in Table 10.

**Fig. 15:**
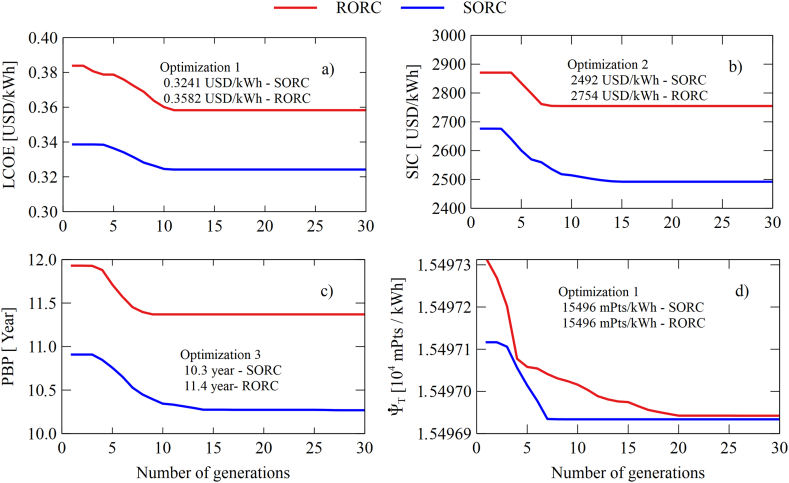
Variations of objective functions in each generation: (a) LCOE, (b) SIC, (c) PBP, and (d) ${\dot{\psi }}_{T}$.

Fig. 15a and b, Figs. 15c and 15d show the evolution of the different objective variables using the advance search of the algorithm for thirty iterations. As for the optimization of the LCOE shown in Fig. 15 shows a reduction of 0.3385 USD/kWh a 0.3241 USD/kWh which represents a decrease of 4.25% for the system SORC and the 6.65% for the system RORC when it comes down from 0.3838 USD/kWh 0.3582 USD/kWh. For the SIC shown in Fig. 15b shows a decrease from 2676 USD/kWh a 2492 USD/kWh for configuration SORC, which represents 6.87%; while for the RORC the minimization was 4.03% As for the investment recovery period (PBP) shown in Fig. 15c shows that the SORC manages to decrease from 10.9 years to 10.25 years which indicates a reduction of 5.86%; while for RORC was 4.69%.

As for the environmental Impact shown in Fig. 15d, the results show that the variation of these parameters has very little influence on the environmental impact. The decrease obtained was only 0.0011%. The explanation for this result is due to the amount of thermal oil used in the construction phase of the equipment. In this study, the tank volume needed to continuously supply the system load ORC in hours of the absence of radiation was 50 m^3^, which is in line with similar investigations in economic terms [25], energetic and exegetical [28] of solar systems -ORC. This implies a volume of 50.5 tons of thermal oil, which means that the thermal oil has the greatest contribution to the environmental impacts of the system, as shown in Table 9. Therefore, variations in the system parameters ORC did not significantly influence this indicator, results that are in closed with [58,59]. Based on the above, to have a realistic estimate of the environmental impact that the system may have depending on the system's operating variables ORC, the system must be redesigned, and the amount of thermal oil in the construction phase must be reduced so that the ${\dot{\Psi }}_{T}$ is much more sensitive before these variations. However, this implies reducing the operability of the system only in hours of radiation and limits the hourly study, which is the focus of this study.

**Table 9:** Maximum and minimum values of decision variables for configurations.

Decision variables	Units	Minimum	Maximum
Pinch point evaporator, Ap	°C	15	35
Condensation temperature, Tcond	°C	40	60
Relation pressure, rp	–	10	30
Collector area, Ac	$m^{2}$	150	200

**Table 10:** Configuration optimization results SORC.

Parameters		Optimized Value			
Symbol	Unit	Optimization 1	Optimization 2	Optimization 3	Optimization 4
rp	–	30	30	30	10.19
Ap	°C	35	35	35	32
$T_{Cond}$	°C	40.43	40.30	40.04	60
$A_{c}$	$m^{2}$	150.27	150.09	150.02	150.4
**LCOE**	USD/kWh	**0.3241**	0.3240	0.3234	0.4074
**SIC**	USD/kWh	2492.05	**2492**	2486	3130
**PBP**	Year	10.26	10.26	**10.3**	12.82
${\dot{\Psi }}_{T}$	mPts/kWh	15498.1	15498.1	15498.52	**15496.96**
$W_{net}$	kW	51.80	51.86	52.0	38.02

**Fig. 16:**
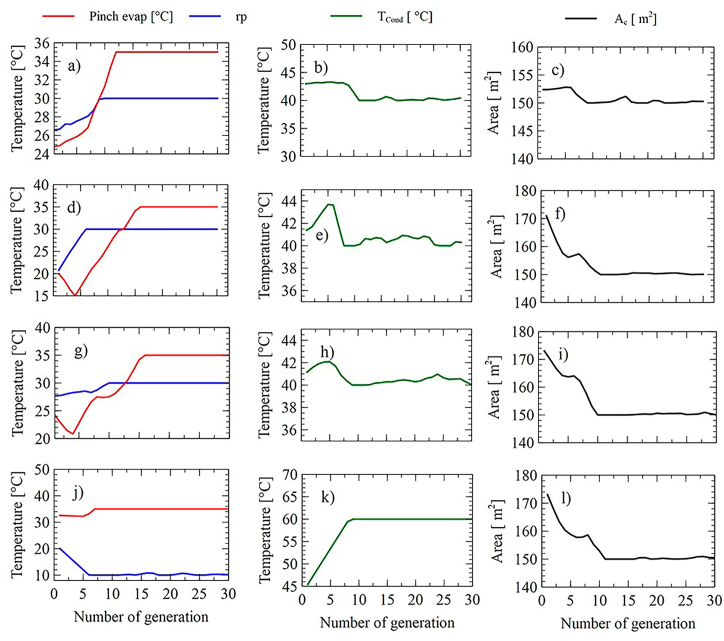
Behavior of decision variables in each generation for configuration SORC for the minimization of LCOE(a-c), SIC(d-f), PBP(g-i), ${\dot{\Psi }}_{T}$ (j–i).

As shown in Fig. 16a, Fig. 16b, c, Fig. 16d, e, Fig. 16f, g, Fig. 16h, i, Fig. 16j, k, and Fig. 16l, the behavior of the decision variables during the search process for the SORC observed. It can be seen that the variables pinch of the evaporator and pressure ratio has a rapid convergence since it does not present variation, which indicates that this is the maximum limit allowed for these variables within the defined search space.

**Fig. 17:**
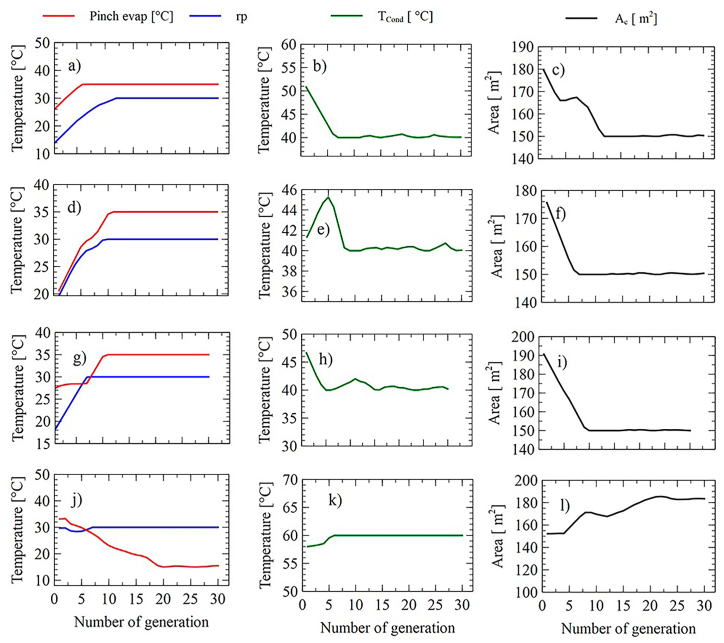
Behavior of decision variables in each generation for the configuration RORC for the minimization of LCOE(a-c), SIC(d-f), PBP(g-i), ${\dot{\Psi }}_{T}$ (j–i).

For the case of condensing temperature, this variable always tended to seek the minimums (Fig. 16b–h). However, when the environmental Impact was minimized, this variable sought its maximum value of 60 °C. In general terms, the minimum values of the LCOE, PBP, and SIC were obtained when the variable pressure ratio and evaporator pinch were at their high limits, while the collector area and condensation temperature near their limits were lower. A similar case was found for the RORC, whose behavior of its variables is shown in Fig. 17a to l.

For this system, the evaporator pinch and pressure ratio variables converge rapidly, between the 10 and 15 iterations.

Table 10 shows the summary of the values obtained from the decision variables, together with the system output variables, in the four optimizations carried out for the system SORC. For optimization 1 (Minimization del LCOE), a value of 0.3241 USD/kWh was obtained. This value, compared to its base condition shown in Table 7, represents a decrease of 11.64%, like the SIC (11.67%) and PBP (12.15). Then optimization 2 (Minimization del SIC) was reduced by 11.67% as well as the LCOE (11.66%), PBP (12.15%). Later in the optimization, 3 (Minimization del PBP) decreased by 11.81%, also LCOE (11.83) and SIC (11.88%). Finally, concerning the minimization of environmental Impact (optimization 4), the reduction obtained was less than 1%. This indicates that these variables' influence was insignificant in reducing this indicator. This is due to the amount of thermal oil required in the construction phase, which greatly contributes to the system's total impact.

Table 11 shows the results obtained for the RORC. It can be seen that in the four optimization scenarios, significant reductions are achieved for LCOE (18.11%), SIC (10.67%), and PBP (11.11%). While for the environmental impact, the reduction was less than 1%.

**Table 11:** Configuration optimization results RORC.

Parameters		Optimized Value			
Symbol	Unit	Optimization 1	Optimization 2	Optimization 3	Optimization 4
rp		30	30	30	30
Ap	°C	35	35	35	15.47
$T_{Cond}$	°C	40.07	40.04	40.20	60
$A_{c}$	$m^{2}$	150.34	150.38	150	183.53
**LCOE**	USD/kWh	**0.3282**	0.3576	0.3578	0.4947
**SIC**	USD/kWh	2750.32	**2754**	2751	3803
**PBP**	Year	11.35	11.35	**11.35**	15.67
${\dot{\Psi }}_{T}$	mPts/kWh	15498.6	15498.6	15498.6	**15496.96**
$W_{net}$	kW	51.98	52	51.91	36.08

Finally, from the results obtained from Table 10, Table 11, it is evident that the values of the decision variables obtained in the optimization of the two configurations did not present significant changes. This leads to infer that values of pressure ratio of 30, evaporator pinch of 35 °C, condensation temperature of 40 °C, and collector area of 150 m^2^ are those that guarantee favorable values in economic terms of the configurations SORC and RORC considered in this study. However, the results obtained from optimization 4 differ from those found in the other optimizations. According to the results of optimizations 1, 2, and 3, there is a reduction in the values of the economic indicators with respect to their base conditions at the expense of the increase in the energy efficiency of the system, which is evidenced in the net power obtained in each optimization (52 kW).

The opposite case is shown in optimization 4, where there is a net power of 36.08 kW. In this case, the data suggest that the reduction of the environmental impact depends on an adequate ratio between the system power and the total solar collector area. Normally, an increase in the system's net power is reflected in an increase in the areas of the heat exchange equipment, which implies higher mass required in both heat exchangers (evaporator, condenser, regenerator, etc.) and turbomachines (turbine, pump). Therefore, during the optimization process, the variation of the environmental impact was favored by reducing the equipment's material requirements and increasing the solar collector's area. However, although a reduction (<1%) in environmental impact was achieved, the loss of energy efficiency at the expense of reduced environmental impact is not competitive. Therefore, for the project's viability, the system should be operated in ranges close to those obtained in optimizations 1, 2, and 3.

## 4 Conclusions

In this work, the energy and economic viability of two configurations were studied ORC powered by a solar collector (flat-plate collector solar) to supply electrical power to a building at Universidad del Atlántico-Colombia. The study consisted of an analysis of the incident solar radiation on the useful heat generation capacity of the collector and the output temperature of the storage tank. Then, an hourly simulation of the consumption, generation, purchase, and sale of energy from the integrated system was carried out solar-ORC with the building for six months representative of the year. Subsequently, economic studies were carried out considering the Relative annual cost (RAB), Levelized cost of energy (LCOE), payback period (PBP), Specific investment cost (SIC), and initial investment cost ($C_{inv}$), these last four were simulated on an hourly basis. Finally, optimization was carried out in which both economic and environmental indicators of the systems were considered.

The results showed that March was the month with the highest accumulated solar radiation compared to the months under consideration, providing a useful heat gain rate in the collector of about 85.11 kW at peak hours.

Concerning the analysis of energy consumption, generation, purchase, and sale, it is concluded that the RORC system was more viable since it presented a higher rate of electricity production concerning the SORC system. This influenced a decrease in the kilowatts per hour purchased in hours in which the demand for energy is greater than that generated by the system.

In economic terms, it is concluded that the configuration SORC showed better performance in terms of relative annual cost (RAB) about the configuration RORC. It is concluded that an evaporator pinch temperature of 35 °C increases RAB the RORC (39604 USD/year) and SORC (39833 USD/kWh). Similarly, an increase in the collection area implies an increase in RAB del SORC (39593 USD/year), and RORC (39364 USD/year), with these variables having the greatest influence on this indicator.

It is concluded that the indicators LCOE, SIC, and PBP tend to increase as the temperature of the source decreases and decreases as the source increases. However, the variations were within a very narrow range allowing optimization to fixed system conditions.

Finally, the application of the optimization algorithm PSO, allowed to reduce of the variables LCOE (11.64%), SIC (11.67%), and PBP (11.81%) for its base condition for the configuration SORC. For the configuration RORC, the reductions were from 18.11%, 10.67%, and 11.11% for the variables LCOE, SIC, and PBP, respectively. However, the reduction in environmental impact for both systems was less than 1% due to the high thermal oil contribution in the system's construction phase.

## Author contribution statement

Guillermo Eliecer Valencia Ochoa: Conceived and designed the experiments; Performed the experiments; Contributed reagents, materials, analysis tools or data; Wrote the paper. Eunice Villicaña Ortiz: Analyzed and interpreted the data; Contributed reagents, materials, analysis tools or data; Wrote the paper. Jorge Duarte Forero: Performed the experiments; Analyzed and interpreted the data; Contributed reagents, materials, analysis tools or data; Wrote the paper.

## Funding statement

This research did not receive any specific grant from funding agencies in the public, commercial, or not-for-profit sectors.

## Data availability statement

No data was used for the research described in the article.

## Additional information

No additional information is available for this paper.

## Declaration of competing interest

The authors declare that they have no known competing financial interests or personal relationships that could have appeared to influence the work reported in this paper.

## Figures and Tables

**Table A.1:** Thermodynamics Properties Solar-SORC configuration.

Point	T [K]	P [kPa]	h [kJ/kg]	s [kJ/kg-K]	s-s0 [kJ/kg -K]	$\dot{E}$ [kJ/s]	$\dot{m}$ [kg/s]
**1**	313.15	7.89	-132.35	-0.38	0.08	0.24	0.457
**2**	313.20	157.85	-132.15	-0.38	0.08	0.32	0.457
**2r**	313.20	157.85	-132.15	-0.38	0.08	0.32	0.457
**2rls**	399.97	157.19	33.03	0.08	0.55	12.60	0.457
**2rvs**	399.97	123.57	370.37	0.95	1.41	49.12	0.457
**T3**	484.75	89.95	526.76	1.28	1.75	74.82	0.457
**T4**	422.74	7.89	424.98	1.33	1.79	22.39	0.457
**T4r**	422.74	7.89	424.98	1.33	1.79	22.39	0.457
**4rvs**	313.15	7.89	271.65	0.91	1.37	9.09	0.457
**T5**	514.75	101.23	488.48	–	1.27	41.95	1.000
**T5a**	514.81	202.46	488.62	–	1.27	41.97	1.000
**5avs**	484.27	199.36	418.63	–	1.08	30.98	1.000
**5ls**	410.13	196.42	262.98	–	0.63	11.38	1.000
**6**	375.03	193.61	196.09	–	0.42	5.34	1.000
**7**	298.15	101.30	104.92	0.37	0.00	0.00	8.841
**7vs**	303.15	77.18	125.82	0.44	0.07	1.53	8.841
**8**	305.05	52.55	133.76	0.46	0.10	2.90	8.841
**9**	453.15	101.23	350.90	–	0.89	21.80	1.000
**9a**	453.22	202.46	350.97	–	0.88	21.80	1.000
**10**	467.08	101.23	380.83	–	0.97	25.65	1.000

**Table A.2:** LCA results for each component of the solar-SORC

Component	Material	Eco-99 [mPts/kg]	Quality [kg]	Y^co^ [mPts]	Y^om^ [mPts]	Y^de^ [mPts]	Y [mPts]
ITC1	Steel	86	200.31	18088	0	481	18949
ITC3	Steel	86	97.86	8407	0	400	8807
TK	Stainless Steel	0.1040^b^	50.00^c^	5,5	0	0.260	6
SC	mixture^a^	0.0172^b^	200.00^c^	3.6	0	0.172	4
T1	Steel	86	1440.49	130076	0	6194	136.270
P1	Steel	86	1.77	160	0	7.6	167
P2	Steel	86	1.96	177	0	8.4	185
P3	Steel	86	1.05	95	0	4.5	99.59
Thermal Oil	73.5% Diphenyl Oxide	46467	50500.00	2409941255	240994125	63357755	2714293134
Fluid	Cyclohexane	2639	257	696508	69651	18311	704561

a The main materials of the solar flat-plate collector are shown in Table 4.

b For the thermal storage tank (TK), the Eco99 coefficient is in units of mPts/m^3^, while for the solar collector (CS) is in mPts/m.^2^.

c For the thermal storage tank (TK) the units are in m^3^and m^2^for the solar collector (CS).

## Nomenclature

$E_{b}$: buying electricity (kW)
$P_{s}$: unit price of selling electricity (USD/kWh)
$E_{s}$: selling electricity (kW)
$P_{b}$: unit price of buying electricity (USD/kWh)
${\dot{Q}}_{col}$: useful heat gain collector (kW)
$C_{inv}$: investment cost (USD)
ITC: heat exchanger
Ap: pinch point evaporator (°C)
rp: relation pressure
$\dot{m}$: mass flow (kg/s)
$\dot{Q}$: heat flow (kW)
e: specific exergy (kJ/kg)
${\dot{Q}}_{ORC}$: load heat orc
h: specific enthalpy (kJ/kg)
$\dot{W}$: power
Nu: Nusselt number
s: specific entropy (kJ/kg-k)
Re: Reynolds number
${\dot{E}}_{D}$: exergy destruction rate (kW)
${\dot{E}}_{Q}$: heat exergy (kW)
$C_{p}$: heat capacity (kJ/kg-k)
$T_{Cond}$: condensation temperature (°C)
$T_{ml}$: long mean temperature difference (°C)
Z: cost
$T_{a}$: ambient temperature (°C)
U: heat transfer coefficient (W/m^2^ K)
A: area (m2)
L: length (m)
W: width (m)
Pr: Prandtl number
Np: plate number
Ac: area collector (m^2^)
UA: overall heat loss coefficient (w/m^2^ °C)
$C_{AP}$: annual production cost (USD)
$C_{TD}$: total depreciable capital
$C_{AD}$: annual depreciation
$S_{annual}$: annual sales revenues direct costs
$C_{MF}$: operating and maintenance cost (USD)
$m_{f}$: maintenance factor
$r_{n}$: nominal scaling ratio
n: plant life time
Greek symbols
η: efficiency
ψ: environmental impact (mPts)
δ: declination solar (°)
φ: latitude angle (°)
$\chi_{d}$: diffuse solar radiation tilt factor
β: collector tilt angle (°)
τ: working hour
$I_{d}$: diffuse solar radiation (W/m^2^)
${\dot{\Psi }}_{T}$: total environmental impact (mPts/kWh)
$\chi_{b}$: beam solar radiation tilt factor
$I_{b}$: beam solar radiation (W/m^2^)
$i_{eff}$: interest rate
$I_{0}$: solar radiation (W/m^2^)

**Abbreviations**
ORC: organic Rankine cycle
SIC: specific investment cost (USD/kWh)
SORC: simple organic Rankine cycle
RAB: relative annual benefit (USD/year)
LCOE: levelized cost of energy (USD/kWh)
RORC: regenerative organic Rankine cycle
PBP: payback period (year)
TK: thermal storage tank
TAC: total annual cost (USD/ year)

**Subscripts**
0: reference condition
T: turbine
out: output
P: pump
CS: collector solar
pre: preheating
Evap: evaporation
Sob: overheating
in: input
to: thermal oil
cv: control volume
wf: working fluid

## References

[1] Barreto R.A. (2018). Fossil fuels, alternative energy and economic growth. Econ. Modell..

[2] Tallaksen J., Johnston L., Sharpe K., Reese M., Buchanan E. (2020). Reducing life cycle fossil energy and greenhouse gas emissions for Midwest swine production systems. J. Clean. Prod..

[3] Evangelisti L., De Lieto Vollaro R., Asdrubali F. (2019). Latest advances on solar thermal collectors: a comprehensive review. Renew. Sustain. Energy Rev..

[4] Gutiérrez-Trashorras A.J., Villicaña-Ortiz E., Álvarez-Álvarez E., González-Caballín J.M., Xiberta-Bernat J., Suarez-López M.J. (2018). Attenuation processes of solar radiation. Application to the quantification of direct and diffuse solar irradiances on horizontal surfaces in Mexico by means of an overall atmospheric transmittance. Renew. Sustain. Energy Rev..

[5] Maurer C., Cappel C., Kuhn T.E. (2017). Progress in building-integrated solar thermal systems. Sol. Energy.

[6] Cancino-Solórzano Y., Villicaña-Ortiz E., Gutiérrez-Trashorras A.J., Xiberta-Bernat J. (2010). Electricity sector in Mexico: current status. Contribution of renewable energy sources. Renew. Sustain. Energy Rev..

[7] Baccioli A., Antonelli M., Desideri U. (2017). Dynamic modeling of a solar ORC with compound parabolic collectors: annual production and comparison with steady-state simulation. Energy Convers. Manag..

[8] Sonsaree S., Asaoka T., Jiajitsawat S., Aguirre H., Tanaka K. (2018). A small-scale solar Organic Rankine Cycle power plant in Thailand: three types of non-concentrating solar collectors. Sol. Energy.

[9] Ashouri, Milad, Ahmadi, Mohammad H., Feidt Michel, Astaraei, Fatemeh Razi. (2017). Exergy and energy analysis of a regenerative organic Rankine cycle based on flat plate solar collectors. Mec. Ind..

[10] Arteconi A., Del Zotto L., Tascioni R., Cioccolanti L. (2019). Modelling system integration of a micro solar Organic Rankine Cycle plant into a residential building. Appl. Energy.

[11] Ustaoglu A., Okajima J., Zhang X.R., Maruyama S. (2019). Assessment of a solar energy powered regenerative organic Rankine cycle using compound parabolic involute concentrator. Energy Convers. Manag..

[12] Ashouri M., Razi Astaraei F., Ghasempour R., Ahmadi M.H., Feidt M. (2015). Thermodynamic and economic evaluation of a small-scale organic Rankine cycle integrated with a concentrating solar collector. Int. J. Low Carbon Technol..

[13] Tiwari D., Sherwani A.F., Kumar N. (2019). Optimization and thermo-economic performance analysis of organic Rankine cycles using mixture working fluids driven by solar energy. Energy Sources, Part A Recover Util Environ Eff.

[14] Garg P., Orosz M.S. (2018). Economic optimization of Organic Rankine cycle with pure fluids and mixtures for waste heat and solar applications using particle swarm optimization method. Energy Convers. Manag..

[15] Desai N.B., Bandyopadhyay S. (2016). Thermo-economic comparisons between solar steam Rankine and organic Rankine cycles. Appl. Therm. Eng..

[16] Spayde E., Mago P.J., Luck R. (2018). Economic, energetic, and environmental performance of a solar powered organic rankine cycle with electric energy storage in different commercial buildings. Energies.

[17] Garcia-Saez I., Méndez J., Ortiz C., Loncar D., Becerra J.A., Chacartegui R. (2019). Energy and economic assessment of solar Organic Rankine Cycle for combined heat and power generation in residential applications. Renew. Energy.

[18] Cioccolanti L., Rajabi Hamedani S., Villarini M. (2019). Environmental and energy assessment of a small-scale solar Organic Rankine Cycle trigeneration system based on Compound Parabolic Collectors. Energy Convers. Manag..

[19] Ramos A., Chatzopoulou M.A., Freeman J., Markides C.N. (2018). Optimisation of a high-efficiency solar-driven organic Rankine cycle for applications in the built environment. Appl. Energy.

[20] Bellos E., Tzivanidis C. (2017). Parametric analysis and optimization of a solar driven trigeneration system based on ORC and absorption heat pump. J. Clean. Prod..

[21] Petrollese M., Cocco D. (2019). Robust optimization for the preliminary design of solar organic Rankine cycle (ORC) systems. Energy Convers. Manag..

[22] Higgo A.R., Zhang T.J. (2015). Characterization of a compact organic rankine cycle prototype for low-grade transient solar energy conversion. Energy Proc..

[23] Li S., Ma H., Li W. (2017). Typical solar radiation year construction using k-means clustering and discrete-time Markov chain. Appl. Energy.

[24] Li S., Li W. (2018). Thermo-economic optimization of solar organic Rankine cycle based on typical solar radiation year. Energy Convers. Manag..

[25] Hajabdollahi H., Ganjehkaviri A., Mohd Jaafar M.N. (2015). Thermo-economic optimization of RSORC (regenerative solar organic Rankine cycle) considering hourly analysis. Energy.

[26] Wang Z., Hu Y., Xia X., Zuo Q., Zhao B., Li Z. (2020). Thermo-economic selection criteria of working fluid used in dual-loop ORC for engine waste heat recovery by multi-objective optimization. Energy.

[27] Wang M., Wang J., Zhao Y., Zhao P., Dai Y. (2013). Thermodynamic analysis and optimization of a solar-driven regenerative organic Rankine cycle (ORC) based on flat-plate solar collectors. Appl. Therm. Eng..

[28] Kerme E.D., Chafidz A., Agboola O.P., Orfi J., Fakeeha A.H., Al-Fatesh A.S. (2017). Energetic and exergetic analysis of solar-powered lithium bromide-water absorption cooling system. J. Clean. Prod..

[29] Bejan A., Tsatsaronis G., Moran M.J. (1995).

[30] Ayub Z.H. (2003). plate heat exchanger literature survey and new heat transfer and pressure drop correlations for refrigerant evaporators. Heat Tran. Eng..

[31] Huang J., Sheer T.J., Bailey-Mcewan M. (2012). Heat transfer and pressure drop in plate heat exchanger refrigerant evaporators. Int. J. Refrig..

[32] García-Cascales J.R., Vera-García F., Corberán-Salvador J.M., Gonzálvez-Maciá J. (2007). Assessment of boiling and condensation heat transfer correlations in the modelling of plate heat exchangers. Int. J. Refrig..

[33] Yan Y.-Y., Lio H.-C., Lin T.-F. (1999). Condensation heat transfer and pressure drop of refrigerant R-134a in a plate heat exchanger. Int. J. Heat Mass Tran..

[34] Ileri̇ A. (1995). Yearly simulation of a solar-aided R22-DEGDME absorption heat pump system. Sol. Energy.

[35] Kotas T.J. (2013).

[36] Goswami D.Y., Kreith F., Kreider J. (2000).

[37] Shams Ghoreishi S.M., Akbari Vakilabadi M., Bidi M., Khoeini Poorfar A., Sadeghzadeh M., Ahmadi M.H. (2019). Analysis, economical and technical enhancement of an organic Rankine cycle recovering waste heat from an exhaust gas stream. Energy Sci \& Eng.

[38] Calise F., Capuozzo C., Carotenuto A., Vanoli L. (2014). Thermoeconomic analysis and off-design performance of an organic Rankine cycle powered by medium-temperature heat sources. Sol Energy.

[39] Zare V. (2015). A comparative exergoeconomic analysis of different ORC configurations for binary geothermal power plants. Energy Convers Manag.

[40] El-Emam R.S., Dincer I. (2013). Exergy and exergoeconomic analyses and optimization of geothermal organic Rankine cycle. Appl Therm Eng.

[41] Mehrpooya M., Ashouri M., Mohammadi A. (2017). Thermoeconomic analysis and optimization of a regenerative two-stage organic Rankine cycle coupled with liquefied natural gas and solar energy. Energy.

[42] Bejan A., Tsatsaronis G. (1996).

[43] Lukawski M. (2009).

[44] Perry S, Perry RH, Green DW, Maloney JO. CHEMICAL ENGINEERS ’ HANDBOOK SEVENTH. (n.d).

[45] Tchanche B.F. (2010).

[46] Han Z. (2017).

[47] Baral S., Kim D., Yun E., Kim K.C. (2015).

[48] Preißinger M., Brüggemann D. (2017). Thermoeconomic evaluation of modular organic rankine cycles for waste heat recovery over a broad range of heat source temperatures and capacities. Energies.

[49] Tchanche B.F. (2010).

[50] Shengjun Z., Huaixin W., Tao G. (2011). Performance comparison and parametric optimization of subcritical Organic Rankine Cycle (ORC) and transcritical power cycle system for low-temperature geothermal power generation. Appl Energy.

[51] Imran M., Park B.S., Kim H.J., Lee D.H., Usman M., Heo M. (2014). Thermo-economic optimization of Regenerative Organic Rankine Cycle for waste heat recovery applications. Energy Convers Manag.

[52] Warren S., Junior S., Daniel L. (2013).

[53] Ding Y., Liu C., Zhang C., Xu X., Li Q., Mao L. (2018). Exergoenvironmental model of Organic Rankine Cycle system including the manufacture and leakage of working fluid. Energy.

[54] Carlsson B., Persson H., Meir M., Rekstad J. (2014). A total cost perspective on use of polymeric materials in solar collectors - importance of environmental performance on suitability. Appl Energy.

[55] (2019). (ISO) IO for S. Environmental Management the ISO 14000 Family of International Standards ISO in Brief ISO and the Environment.

[56] Kennedy J., Eberhart R. (1995). Particle swarm optimization. Int Conf Neural Networks.

[57] Ochoa G.V., Prada G., Duarte-Forero J. (2020). Carbon footprint analysis and advanced exergo-environmental modeling of a waste heat recovery system based on a recuperative organic Rankine cycle. Journal of Cleaner Production.

[58] Herrera-Orozco I., Valencia-Ochoa G., Duarte-Forero J. (2021). Exergo-environmental assessment and multi-objective optimization of waste heat recovery systems based on Organic Rankine cycle configurations. Journal of Cleaner Production.

[59] Duarte-Forero J., Obregón-Quiñones L., Valencia-Ochoa G. (2021). Comparative analysis of intelligence optimization algorithms in the thermo-economic performance of an energy recovery system based on organic rankine cycle. Journal of Energy Resources Technology.

